# Triploid Cyprinid Fish (TCF) Under *Aeromonas* sp. AS1-4 Infection: Metabolite Characteristics and In Vitro Assessment of Probiotic Potentials of Intestinal *Enterobacter* Strains

**DOI:** 10.3390/biology14111485

**Published:** 2025-10-24

**Authors:** Xu-Ying Kuang, Qin-Yang He, Zi-Xuan Fang, Zhuang-Wen Mao, Ming-Zhu Huang, Zi-Le Qin, Jie Peng, Yu-De Wang, Sheng-Wei Luo

**Affiliations:** 1 Engineering Research Center of Polyploidy Fish Reproduction and Breeding of the State Education Ministry, College of Life Science, Hunan Normal University, Changsha 410081, China; kuangxy2025@shanghaitech.edu.cn (X.-Y.K.); 202230233020@hunnu.edu.cn (Q.-Y.H.); 2025202040090@whu.edu.cn (Z.-X.F.); 2025023034@m.scnu.edu.cn (Z.-L.Q.); 17369605790@163.com (J.P.); 2 Fish Healthy Breeding Sub-Center of State Key Laboratory of Freshwater Fish Developmental Biology, Hunan Provincial Key Laboratory of Nutrition and Quality Control of Aquatic Animals, Department of Biological and Chemical Engineering, Changsha University, Changsha 410022, China; z20200820@ccsu.edu.cn; 3 National R&D Center for Freshwater Fish Processing, Jiangxi Normal University, Nanchang 330022, China; huangmingzhu12@163.com

**Keywords:** hybrid fish, metabolomics, fish pathogens, probiotic isolation

## Abstract

**Simple Summary:**

Our research found that *Aeromonas* sp. AS1-4 infection could induce severe oxidative damage in the liver and spleen of TCFs, along with a significant alteration of amino acid metabolism. Following this, we identified three probiotic isolates displaying anti-pathogenic capability and potential traits in vitro. This study provided a valuable insight into exploring the pathogenic mechanism and guiding the probiotic application in aquaculture practices.

**Abstract:**

The majority of *Aeromonas* strains are opportunistic pathogens for both humans and animals, causing a variety of diseases and posing a considerable risk to their health. In the current study, triploid cyprinid fish (TCF) were infected with a novel pathogenic *Aeromonas* sp. AS1-4 for pathological analysis. TCFs receiving *Aeromonas* sp. AS1-4 challenge exhibited oxidative damage in the liver and spleen, along with significant changes in immune-related gene expressions. Metabolomics assay indicated that strain AS1-4 challenge may exhibit a significant impact on metabolic processes of amino acids, with methylsuccinic acid (MSA) identified as vital biomarker. Following that, three potential probiotics designated *Enterobacter* strains fkY27-2, fkY84-1 and fkY84-4 were isolated from the intestine of TCFs, displaying excellent safety characteristics. In addition, intestinal *Enterobacter* strains exhibited multiple probiotic traits, including high degrees of hydrophobic activity, aggregation performance, biofilm-forming activity (BFA) and nutrient decomposing ability. Moreover, these probiotic isolates markedly coaggregated with *Aeromonas* sp. AS1-4 and *Edwardsiella tarda* 1l-4 and then suppressed their pathogenic biofilm-forming abilities, along with possessing robust antioxidant potential against various free radicals. These findings may provide valuable insights into metabolic response in fish post infection and health management in aquaculture.

## 1. Introduction

Crucian carp (*Carassius auratus*) occupies a prominent position in Chinese aquaculture [1], but deterioration of aquaculture environment may severely increase the susceptibility of fish to pathogenic infection [2]. Given this situation, a novel economic fish species with high disease resistance is urgently needed. Hybridization may facilitate the generation of novel phenotypes in hybrid offspring, which is widely considered as the practical strategy for large-scale generation of economic fish with improved traits [3]. Allotetroploid cyprinid fish (4N = 200) were generated by intergeneric hybridization of red crucian carp (*Carassius auratus* red var, 2N = 100, ♀) and common carp (*Cyprinus carpio*, 2N = 100, ♂) [4]. Then, triploid cyprinid fish (TCF, 3N = 150) were obtained by crossing of red crucian carp (2N = 100, ♀) and allotetroploid fish (4N = 200, ♂) [5]. In our previous findings, TCFs exhibited a higher resistance against *Aeromonas hydrophila* infection compared with that of RCC, suggesting that TCFs may act as the triploid hybrid animal model to evaluate the pathological mechanism of Aeromonas strains [6].

When referring to immune properties, innate immunity of fish encompasses complement cascades and pattern recognition receptors (PRRs), which may act as initial barrier against pathogenic incursion [7]. Nevertheless, a certain number of aggressive bacteria are able to circumvent the host’s immune detection and then dampen antibacterial reaction [8]. For instances, *Edwardsiella piscicida* challenge can induce severe pathology and mortality in catfish [9]. Our previous study demonstrated that grass carp receiving gut infection with *Aeromonas hydrophila* L3-3 displayed intense lesions in the intestine and liver, along with a significant suppression of antioxidant capacity [10]. Furthermore, *Aeromonas* strains can secrete toxins with distinct thermal stability, which are closely linked to multiple pathological conditions, prominently manifesting as watery diarrhea [11], while *Edwardsiella* strains are among etiologic agents that can induce edwardsiellosis in commercial fish species [12]. Despite the fact that the employment of a variety of drugs effectively mitigates the risk of disease outbreaks in aquaculture, overusing them tends to result in escalating antibiotic resistance among natural microbes [13]. Prolonged exposure to antibiotics might promote intestinal dysbiosis in fish, which can weaken the host’s immune response to invading pathogens [14]. While probiotics cannot replace antibiotics in treating acute infections, they serve as an effective preventive measure that reduces the frequency and intensity of disease outbreaks in aquaculture.

Probiotics are a scientific designation for microbes that aid in maintaining gut microbial balance and offer health benefits to both humans and animals [15]. Recently, an increasing number of probiotics have been isolated from the digestive tract, which can improve nutritional absorption and modulate the host’s immune response [16]. As commonly understood, the composition of intestinal flora is closely associated with the combined influence of genetic factors, nutritional substances and environmental factors [17]. Among known probiotic species, *Lactobacillus* and *Bacillus* strains have been widely investigated for desirable traits [18]. *Enterobacter* strains were widely considered as the opportunistic pathogens associated with human diseases [19]. Lately, *Enterobacter asburiae* C28 showed beneficial effects on the height of intestinal plica and promoted nutrient absorption in crucian carp [20]. Additionally, *Enterobacter asburiae* E7 could induce immune-related gene expressions in common carp and elevate resistance against *Aeromonas veronii* infection [21]. However, there is a scarcity of reports on the desirable traits for probiotic potential and genomic characteristics of *Enterobacter* strains isolated from the intestine of TCFs.

The primary objectives of this investigation were to unravel the alteration of redox and metabolic status after strain AS1-4 challenge. Following that, three *Enterobacter* strains isolated from the intestine of TCFs were subjected to genomic identification by whole genome sequencing (WGS). In addition, probiotic properties and inhibition of fish pathogens were investigated in vitro. This study may provide a novel perspective on the metabolic response and probiotic potentials of bacterial isolates from TCFs.

## 2. Materials and Methods

### 2.1. Ethics Approval

All procedures, including the care and use of experimental fish, were approved by the Animal Care and Use Committee of Hunan Normal University (Changsha City, Hunan province, China) and the Technical Committee for Laboratory Animal Sciences of the Standardisation Administration of China (SAC/TC281).

### 2.2. Determination of Pathological Traits Induced by Aeromonas *sp.* AS1-4

#### 2.2.1. Fish Preparation and Immune Challenge Assay

Healthy TCFs (average body length of 19.4 ± 1.19 cm) were sourced from an aquaculture base in Wangcheng District, Changsha, China. Fish were transferred to a plastic aquarium filled with clean freshwater to undergo a two-week acclimation. Fish were fed twice a day throughout the acclimation period. Feeding was halted 24 h prior to infection assay. Moreover, strict control of water quality was implemented to prevent any pathogenic contamination during both the acclimation phase and immune challenge procedures.

Strain AS1-4 was cultivated for 24 h at 30 °C, centrifuged at 10,000× *g* for 15 min and then resuspended in 1 × PBS (pH 7.3) before use. Based on our previous study [22], fish intraperitoneally infected with strain AS1-4 (1 × 10^7^ CFU/mL, 100 μL) were used as the infection group, while fish in the control group were injected with the same volume of sterile PBS. Tissues were collected at 24 h post-infection. Samples were promptly frozen in liquid nitrogen and then stored at −80 °C.

#### 2.2.2. Evaluation of Biochemical Metrics

After homogenization on ice, protein contents of isolated tissues were quantified by BCA method. Based on previous studies, redox-related enzymatic activities were measured [10]. Catalase (CAT) activity was evaluated using a CAT kit (OD_405_, Nanjing Jiancheng Bioengineering Institute, Nanjing, China). Glutathione peroxidase (GPx) activity was evaluated using a GPx kit (OD_340_, Beyotime Biotechnology, Shanghai, China). Glutathione reductase (GR) activity was evaluated using a GR kit (OD_412_, Nanjing Jiancheng Bioengineering Institute, Nanjing, China). Lactate dehydrogenase (LDH) activity was evaluated using a LDH kit (OD_490_, Beyotime Biotechnology, Shanghai, China). Monoamine Oxidase (MAO) activity was evaluated using a MAO kit (OD_242_, Nanjing Jiancheng Bioengineering Institute, Nanjing, China). Succinate dehydrogenase (SDH) activity was evaluated using a SDH kit (OD_600_, Nanjing Jiancheng Bioengineering institute, Nanjing, China). The experiments contained three individual biological repeats.

#### 2.2.3. Metabolite Characteristics and Annotation by Metabolomics

Metabolite features in the liver were analyzed using LC-MS/MS approach (Shanghai Kehua Bio-Engineering Co., Ltd., Shanghai, China). Once the proteins were precipitated and removed from the samples using an organic solvent, the remaining residues were re-extracted with 70% methanol, and then the resulting filtrates were dried using a vacuum centrifugal concentrator (Ningbo Scientz Biotechnology Co., Ltd., Ningbo, China). Dried samples were then analyzed through a combination of UPLC and QTOF-MS in both positive and negative ion modes (scan range 50–1000 *m*/*z*). L-2-chlorophenylalanine was used as internal standard. The binary gradient system consisted of water (containing 0.1% formic acid, *v*/*v*) and acetonitrile (containing 0.1% formic acid, *v*/*v*). The chromatographic conditions were set as follows: column temperature maintained at 40 °C, flow rate of 0.4 mL/min and injection volume of 2.0 μL. Throughout the analytical run, QC was injected at consistent time intervals. XCMS program was employed to compute raw data. Once metabolic results were identified against the metabolomic database, PLS-DA and OPLS-DA models were further utilized for analysis. The criteria for screening differential metabolites (DMs) were defined as follows: variable importance in projection (VIP) ≥ 1, *p*-value < 0.05, and fold change ≥ 1.5 or fold change ≤ 0.67, and the identified DMs meeting these criteria were further subjected to Kyoto Encyclopedia of Genes and Genomes (KEGG) [23]. Each group in metabolome employed six individual biological replicates.

#### 2.2.4. RNA Isolation and Quantitative Real-Time PCR (qPCR) Analysis

Total RNA isolation was carried out using FastPure cell/tissue total RNA isolation Kit V2 (Vazyme Biotech, Nanjing, China). In order to preclude genomic DNA contamination, obtained RNA was subsequently incubated with DNAase [24]. Once RNA quality had been determined, cDNA synthesis (1000 ng RNA/reaction) was executed by using a reverse transcription kit (Monad, Shanghai, China).

For detection of immune-related gene expressions, relative patterns of cytokines (cxcl10, ccl2 and il8), cluster of differentiation molecules (cd22, cd28, cd3e, cd4 and cd81), heat-shock proteins (hsp70 and hsp90α), Fc epsilon receptor Ig (fcer1g), immunoglobulin heavy constant mu (ighm), natural killer lysin (NK-lysin) and complement component 1q (c1q) were explored. For validation of metabolic gene expressions, relative patterns of low molecular weight phosphotyrosine protein phosphatase (lmptp), fructose-1,6-bisphosphatase (fbp), guanidinoacetate N-methyltransferase (gamt), liver-type fatty acid-binding protein (l-fabp), 7-Dehydrocholesterol Reductase (dhcr7) and NADH-cytochrome b5 reductase 2 (cyb5r2) were investigated. In addition, 18S rRNA was selected as reference gene.

In line with manufacturer’s instructions, qPCR reaction comprised 10.0 μL of SYBR green mix, 2.0 μL of cDNA template, 0.5 μL of each primer and 7.0 μL of ddH_2_O. After qRT-PCR results verified by melting curve analysis, and data were calculated using 2^−ΔΔCt^ method [25]. The primer sequences are presented in Table S1. Each group consisted of three individual biological replicates.

### 2.3. Probiotic Potentials of Bacteria Isolates from TCFs

#### 2.3.1. Bacterial Identification

Bacterial isolation procedures followed the previously reported protocol [26]. Healthy TCFs (approximately 25.41 ± 3.15 g, N = 10) were collected from a fishing base (Changsha City, China) and acclimated as mentioned above. Moreover, strict control of water quality was implemented to prevent any pathogenic contamination during both the acclimation phase and immune challenge procedures. After enrichment, intestinal homogenates were cultured on Luria–Bertani (LB) agar plates (pH 7.3) at 30 °C for 24 h. Individual bacterial colonies were selected based on distinct morphologies [27]. Among bacterial isolates, three probiotic isolates displayed strong bacteriostatic potential against fish pathogens. The 16S rRNA gene sequences of strains fkY27-2, fkY84-1 and fkY84-4 were submitted to GenBank (OQ927098, OQ927182, OQ927191). Phylogenetic tree analysis was constructed using MEGA 7.0 software, employing the neighbor-joining (N-J) algorithm for analysis.

#### 2.3.2. Genomic Characteristics

Genomic features of strains fkY27-2, fkY84-1 and fkY84-4 were investigated by WGS, leveraging a hybrid approach that integrated Nanopore and Illumina sequencing (Tsingke biotech, Beijing, China). Adhering to the established protocol, bacterial DNA extraction and purification were executed using the QIAGEN Genomic-tip kit (QIAGEN GmbH, Hilden, Germany) [28]. Post data filtration, the obtained long reads were assembled with Canu v1.5 software. Prodigal v2.6.3 was implemented to delineate the genomic architecture, while Circos v0.66 facilitated the construction of a circular genomic map. For in-depth functional analyses, a suite of bioinformatics resources was deployed. Genomic islands (GIs) were pinpointed via IslandPath-DIOMB. Carbohydrate-active enzymes were cataloged using the CAZy database. Virulence genes (VGs, Identity > 85% and E-value < 1 × 10^−15^) were characterized against VFDB database [29]. Transporters were identified with TCDB database. Core biosynthetic gene clusters (cBGCs) in secondary metabolite regions (SMRs) were profiled by antiSMASH 7.0. Following that, the assembled genomes of strains AS1-4, fkY27-2, fkY84-1 and fkY84-4 were deposited in NCBI.

#### 2.3.3. Determination of Antibiotic Resistance

Strains fkY27-2, fkY84-1 and fkY84-4 (1 × 10^8^ CFU/mL) were spread onto LB agar plates. Following that, distinct antibiotic discs were precisely positioned on the plates. Subsequently, the plates were incubated at 30 °C overnight. In accordance with previous study, the dosage of antibiotics and the criteria for evaluating the inhibition zone diameter (IZD) were established [27]. Each group consisted of three biological replicates.

#### 2.3.4. Determination of Hemolytic Ability

Strains fkY27-2, fkY84-1 and fkY84-4 (1 × 10^8^ CFU/mL) were incubated on 5.0% (*v*/*v*) goat blood agar plate at 30 °C for 24 h, whose hemolytic types were determined based on the formation of hemolytic zone. This experiment was conducted with three biological replicates.

#### 2.3.5. Assessment of Stress Resistance

The effects of different variables, such as acid challenge, bile salt (BS) exposure, lysozyme (LZM) stimulation and NaCl salinity exposure, on the survival of strains fkY27-2, fkY84-1 and fkY84-4 were analyzed at 3 h, 12 h and 24 h (2.0% inoculum, OD_600_ = 0.6, 30 °C). In brief, the effect of acid challenge on probiotic survival was analyzed by value changes of 2.5, 3.5 and 4.5 [30]. BS’s effect on probiotic survival was analyzed by altering ratios of 0.1%, 0.3% and 0.5% [31]. LZM’s effect on probiotic survival was analyzed by concentration change of 50 μg/mL, 100 μg/mL and 200 μg/mL [32]. The effect of NaCl salinity on probiotic survival was analyzed by value changes of 2.0%, 4.0% and 6.0% [33]. LB broth without treatment was used as the control group. OD_600_ values of test group, control group and blank group were shown as A_t_, A_c_ and A_0_, respectively. Survival rate (SR) was quantified as below: SR = (A_t_ − A_0_)/(A_c_ − A_0_) × 100%. This experiment was conducted with three biological replicates.

#### 2.3.6. Aggregation Analysis

Strains fkY27-2, fkY84-1 and fkY84-4 were cultured in LB medium overnight and then resuspended with an OD_600_ value of 0.8 (A_0_) in PBS. For autoaggregation (AAg) assay, 4.0 mL of probiotic suspensions were incubated at 30 °C for 24 h, then OD_600_ values were recorded as A_1_. For coaggregation (CAg) assay, *Aeromonas* sp. AS1-4 and *Edwardsiella tarda* 1l-4 were used as fish pathogens whose OD_600_ values were adjusted to 0.7 (A_fp_) before use. Afterwards, 2.0 mL of probiotic suspensions were incubated with an equivalent volume of fish pathogens at 30 °C for 24 h, then their OD_600_ values were shown as A_2_. Then, AAg ratio and CAg ratio were quantified as follows: AAg = 1 − A_1_/A_0_ × 100% and CAg = (1 − A_2_)/[(A_fp_ + A_0_)/2] × 100%. This experiment was conducted with three biological replicates.

#### 2.3.7. Hydrophobicity Assay

Strains fkY27-2, fkY84-1 and fkY84-4 were cultured in LB medium overnight and then resuspended with an OD600 value of 0.8 (A_0_) in PBS. 3.0 mL of probiotic suspensions were incubated with 1.0 mL of chloroform or xylene for 20 min and then the organic phase was removed. Afterwards, obtained liquids were diluted and their OD_600_ values were recorded as A_h_. Hydrophobicity ratio was quantified as follows: Hydrophobicity = (A_0_ − A_h_)/A_0_ × 100%. This experiment was conducted with three biological replicates.

#### 2.3.8. Biofilm Assay

Strains fkY27-2, fkY84-1 and fkY84-4 were cultured in LB medium overnight and then resuspended with an OD600 value of 0.8 (A_0_) in PBS. 200.0 μL of probiotic strains (1 × 10^8^ CFU/mL) were added into 96-well plates at 30 °C for 24 h. After fixation and PBS wash, bacterial colonies were stained with crystal violet and their OD_570_ values were shown as A_bfa_. OD_570_ value of LB medium alone was shown as A_c_. Then, AAg ratio, CAg ratio, hydrophobicity ratio and BFA ratio were quantified as follows: BFA ratio = A_bfa_/A_c_. When ratios > 1, probiotics were biofilm-positive strains. This experiment was conducted with three biological replicates.

#### 2.3.9. Inhibitory Effect of Probiotics on Biofilm Formation of Fish Pathogens

Culture medium (CM) of the probiotic strain was harvested after 72 h of incubation. Then, CM was sterilized by filtration sterilization and used as cell-free supernatant (CFS). In brief, *Aeromonas* sp. AS1-4 and *E. tarda* 1l-4 (1 × 10^6^ CFU/mL) were used as fish pathogens. A total of 10.0 μL of fish pathogens were cultured with 190.0 μL of CM or CFS at 30 °C for 24 h. After fixation, bacterial colonies were stained with crystal violet. OD_570_ values of pathogenic biofilm treated with CFS and CM were recorded as A_CFS_ and A_CM_, respectively. In addition, OD_570_ values of pathogenic biofilm without treatment were recorded as A_0_. Biofilm inhibition (BI) ratio was quantified as follows: BI ratio = (1 − A_CFS (CM)_/A_0_) × 100%. This experiment was conducted with three biological replicates.

#### 2.3.10. Detection of In Vitro Degradation Performance

The supernatants of probiotic strains were harvested after 48 h of incubation. For protein degradation assay, casein solution (CS, 10.0 mg/mL) was used as protein substrate. A total of 0.5 mL of probiotic supernatants were added to an equivalent volume of CS whose OD absorbance was quantified at 500 nm by Lowry method. The supernatant of CS alone was used as the control group. Results were shown as U of protease (PRS) activity per litter of probiotic supernatant, where 1.0 U of PRS activity is defined as the amount of enzyme degrading 1.0 μg CS per min. This experiment was conducted with three biological replicates.

For starch degradation analysis, soluble starch (SS, 0.4 mg/mL) was used as starch substrate. A total of 0.5 mL of probiotic supernatants were added to equivalent volume of SS, whose OD absorbance was measured at 660 nm by iodine colorimetric method. The supernatant of SS alone was used as the control group. Results were shown as U of amylase (AMS) activity per milliliter of probiotic supernatant, where 1.0 U of AMS activity is defined as the amount of enzyme degrading 10.0 mg SS per min. This experiment was conducted with three biological replicates.

For lipid degradation analysis, triglyceride (TG, 454.0 μmol/L) was used as lipid substrate. 50.0 μL of probiotic supernatants were added to 1.95 mL of TG, whose OD absorbance was measured at 420 nm by turbidimetric method. Results were shown as U of lipase (LPS) activity per milliliter of probiotic supernatant, where 1.0 U of LPS activity is defined as the amount of enzyme degrading 1.0 μmol TG per min. This experiment was conducted with three biological replicates.

#### 2.3.11. Detection of Antioxidant Ability

Strains fkY27-2, fkY84-1 and fkY84-4 were cultured in LB medium at 30 °C. Afterwards, probiotic strains were collected during logarithmic growth period. Then, probiotic strains (1 × 10^8^ CFU/mL) were exposed to tert-butyl hydroperoxide (TBHP, 1.55 mmol/L) and H_2_O_2_ (0.35 mmol/L) for 32 h. After wash with PBS, probiotic strains were used for detection of free radical scavenging (FRS) ability. For superoxide anion radical scavenging (SARS) ability, OD absorbance was measured at 530 nm by using a SARS activity kit (Solarbio, Beijing, China). For DPPH radical scavenging (DPPHS) ability, OD absorbance was measured at 517 nm by using a DPPHS activity kit (Jiancheng Bioengineering institute, China). FRS ability was calculated as follows: FRS activity = [(1 − (A_t_/A_c_)] × 100%. Wherein, A_t_ represented the absorbance of the test group, while A_c_ represents that of the control group. This experiment was conducted with three biological replicates.

### 2.4. Statistical Analyses

The above experimental results were subjected to one-way ANOVA by using SPSS 17.0 software. If the analytical level of *p*-value reaches less than 0.05, results were statistically significant. In addition, results of probiotic features were calculated by principal component analysis (PCA).

## 3. Results

### 3.1. Expression Profiles of Immune-Related Genes

To evaluate the immune response triggered by strain AS1-4 infection, the patterns of vital immune-associated genes were determined in the liver and spleen of TCFs post-infection. In Figure S1A, relative expressions of *il8*, *cxcl10*, *fcer1g*, *cd81*, *ighm*, hsp70, *hsp90α*, *c1q* and *cd22* increased by 87.9-, 77.9-, 53.3-, 22.9-, 11.6-, 10.1-, 5.1-, 5.3- and 2.2-fold, respectively, in the liver of TCFs infected with strain AS1-4, while expression profiles of *ccl2*, cd4, *cd3e*, *cd28* and *NK-lysin* declined sharply. TCFs receiving strain AS1-4 showed a 3.9-, 2.6-, 2.3-, 2.1- and 1.4-fold elevation of *cd22*, *NK-lysin*, *ccl2*, *cd4* and *cd3e*, respectively, in the spleen, while expressions of *cd28*, *c1q*, *hsp90α*, *hsp70*, *ighm*, *cd81*, *fcer1g*, *cxcl10* and *il8* dropped dramatically (Figure S1B). In Figure S1C, PCA method was employed to measure variations in expression profiles of immune-related genes in liver and spleen. It was noted that expressions of cytokines, HSPs and immune regulators were situated in the second quadrants, which showed a horizontal mirror-image correspondence with the majority of CD genes.

### 3.2. Evaluation of Antioxidant Property After Strain AS1-4 Infection

**Figure 1:**
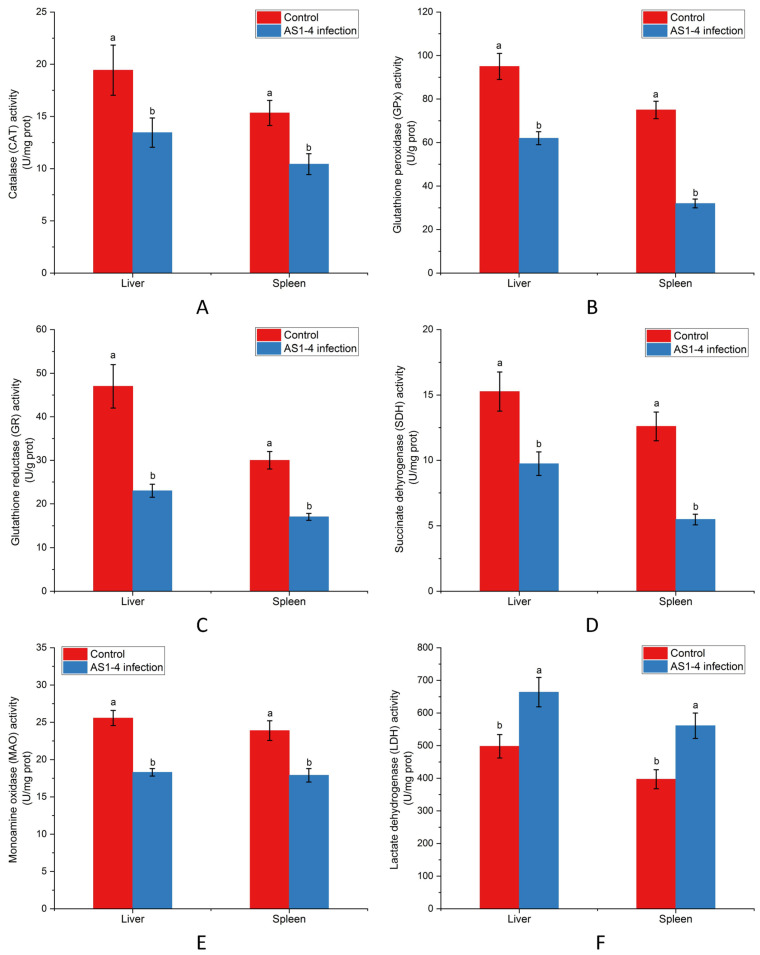
Assessment of redox features in liver and spleen of TCFs after AS1-4 infection. (**A**) CAT activity. (**B**) GPx activity. (**C**) GR activity. (**D**) SDH activity. (**E**) MAO activity. (**F**) LDH activity. Calculated data (mean ± SD) with different letters were significantly different (*p* < 0.05) among groups. The experiments contained three biological repeats.

In Figure 1A, TCFs infected with strain AS1-4 displayed a 1.44- and 1.47-fold decrease in CAT activities in the liver and spleen, respectively. GPx activities decreased by 1.53- and 2.34-fold in the liver and spleen, respectively, of TCFs post infection (Figure 1B). Similarly, 2.04- and 1.76-fold declines of GR activities were recorded in the liver and spleen, respectively (Figure 1C). Meanwhile, 1.56- and 2.30-fold decreases in SDH activities were observed in the liver and spleen, respectively, following strain AS1-4 challenge (Figure 1D). As shown in Figure 1E,F, 1.40- and 1.33-fold decreases in MAO activities were observed in the liver and spleen, respectively, upon infection, whereas LDH activities the in liver and spleen rose by 1.33- and 1.41-fold, respectively.

### 3.3. Determination of Metabolic Characteristics

In order to elucidate vital metabolic indicators in the liver of TCFs after strain AS1-4 challenge, a total of 3412 primary metabolites were identified by LC-MS/MS approach. As depicted in Figure S2A, “Amino acid and its metabolites” contained the highest metabolic proportion (17.94%) among subclasses, succeeded by “Benzene and substituted derivatives” (16.32%) and “Heterocyclic compounds” (13.16%). Isobutyric acid displayed the highest concentration among identified metabolites, succeeded by methylsuccinic acid (MSA), while ganoderic acid A emerged as the metabolite with the least amount (Figure S2B). In addition, expression patterns of crucial metabolic indicators were investigated in Figure S2C. Increases of 2.77-, 11.02- and 3.48-fold of *lmptp*, *dhcr7* and *cyb5r2* were detected in the liver of TCFs after strain AS1-4 infection, while gene expressions of *fbp*, *gamt* and *l-fabp* declined dramatically.

**Figure 2:**
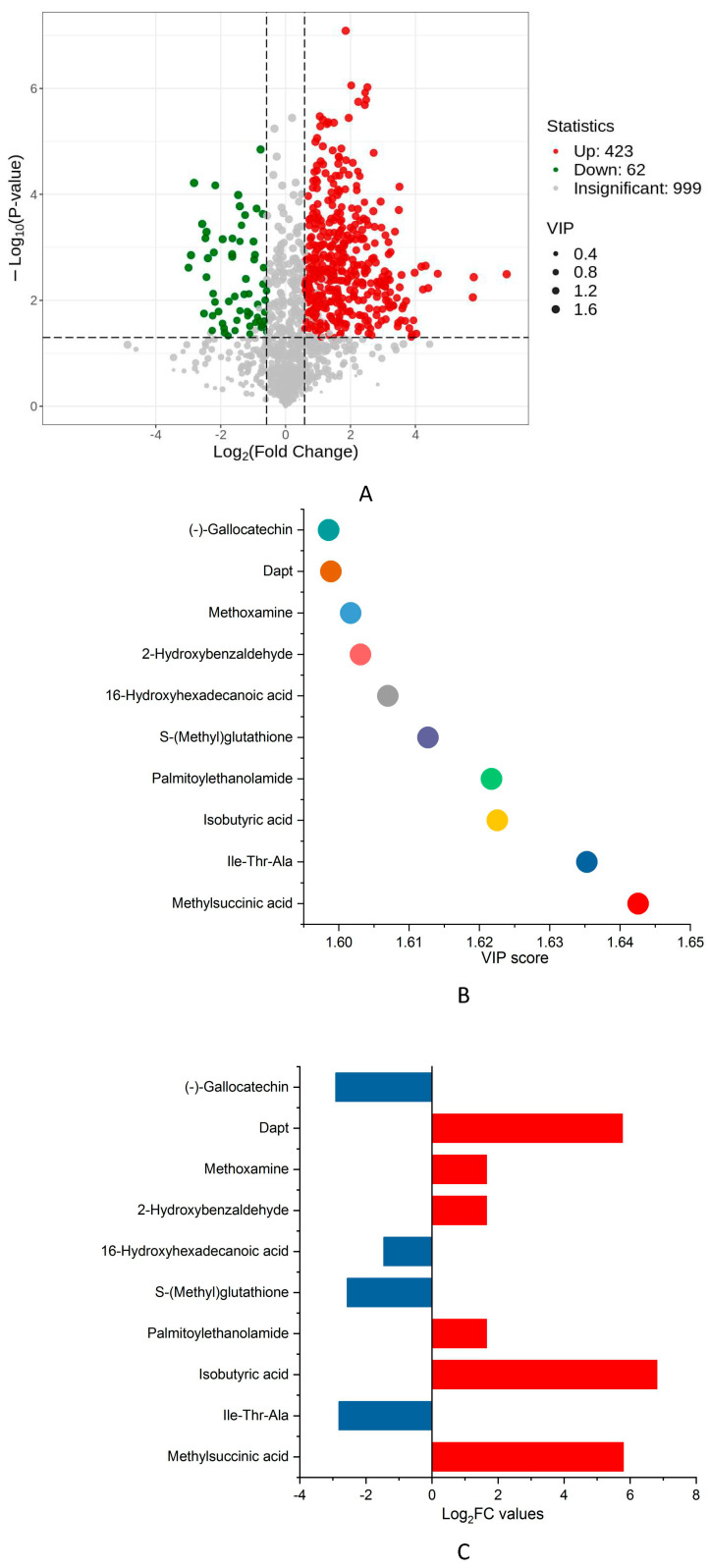
DMs identification. (**A**) DM Numbers by volcano plot. Red spots represented increased DMs, whereas green spots represented decreased DMs. (**B**) VIP scores of TOP10 DMs. (**C**) Log2FC values of TOP10 DMs.

In Figure S2D–G, the clear distinctions in metabolic manifestations among different groups were examined by PLS-DA and OPLS-DA analyses. Based on OPLS-DA model, S plot analysis was employed to determine the distinction of differential metabolites (DMs) by computing VIP threshold (Figure S2H). In Figure 2A, a total of 423 upregulated DMs were recorded in the liver of TCFs after strain AS1-4 challenge, while 62 DMs experienced a notable decline. Methylsuccinic acid (MSA) displayed the highest levels of VIP scores, followed by Ile-Thr-Ala, while DMs with high log2FC values included isobutyric acid, Dapt and MSA (Figure 2B,C).

**Figure 3:**
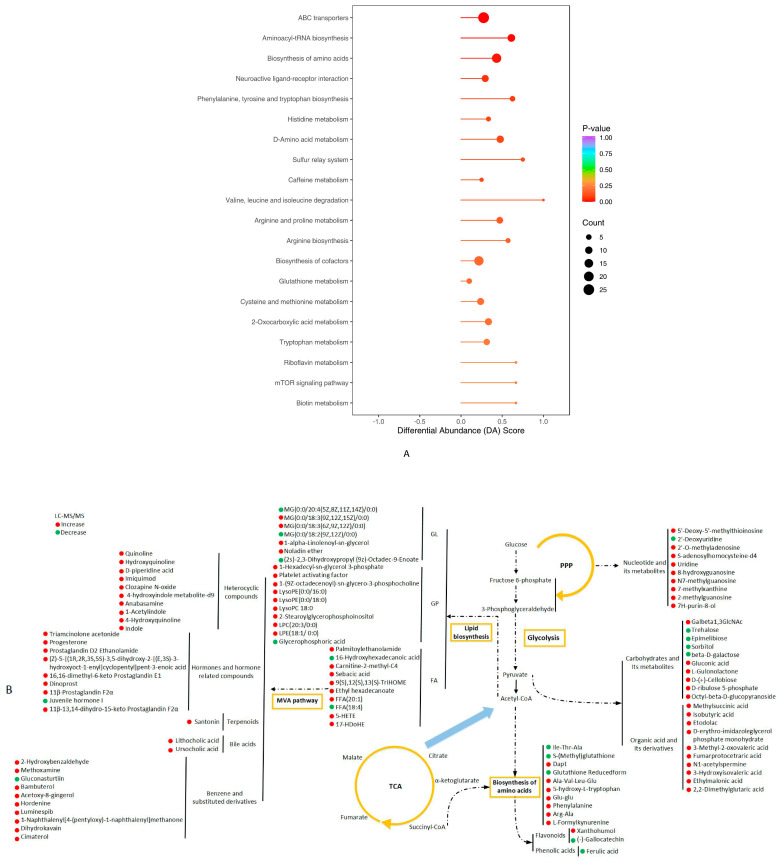
Metabolic signals in the liver of TCFs after strain AS1-4 infection. (**A**) KEGG pathway of annotated DMs with the calculation of differential abundance (DA) score. Enriched DMs were labeled from red to purple based on *p*-values. (**B**) Network analysis of pivotal DMs in liver of TCFs after strain AS1-4 infection. Increased DMs were indicated by red circles, while decreased DMs were indicated by green circles.

In Figure 3A, TOP20 KEGG subgroups displayed the upregulatory trend, and “ABC transports” possessed the greatest number of annotated DMs. In addition, the vast majority of DMs experienced a striking rise in pentose phosphate pathway (PPP), lipid biosynthesis, mevalonic acid (MVA) pathway, carbon metabolism and amino acid biosynthesis (Figure 3B).

### 3.4. Genomic Identification of Intestine-Derived Probiotic Isolates

**Figure 4:**
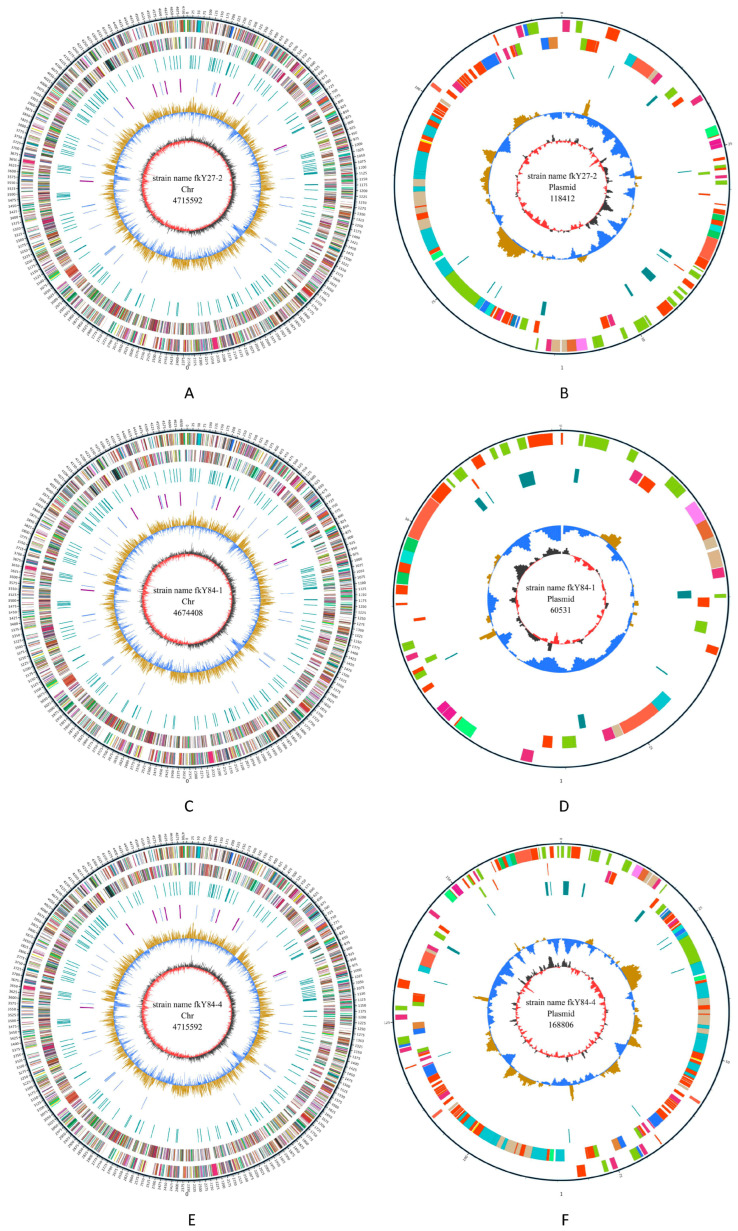
Circular genome map of bacterial isolates. (**A**,**B**) Complete genome of Chr and plasmid in strain fkY27-2. (**C**,**D**) Complete genome of Chr and plasmid in strain fkY84-1. (**E**,**F**) Complete genome of Chr and plasmid in strain fkY84-4.

In this investigation, strains fkY27-2, fkY84-1 and fkY84-4 manifested as the milky-white and circular colony with a moist surface (Figure S3A–C). Phylogenetic analysis delineated three strains of probiotic isolates that displayed a close evolutionary relationship to other *Enterobacteria* strains (Figure S3D). Furthermore, strains fkY27-2, fkY84-1 and fkY84-4 displayed low resistance to the majority of the tested antibiotics (Figure S3E). In addition, the chromosome genome of strain fkY27-2 displayed a high similarity to those of strains fkY84-1 and fkY84-4, while the plasmid of strain fkY84-4 was larger in size and contained more gene sets than those of the other two strains (Figure 4A–F and Table S2). However, plasmid structures of three probiotic isolates lacked several crucial components, such as ncRNA, CRISPR and gene cluster (Table S2). Furthermore, the category of glycoside hydrolases in strains fkY27-2, fkY84-1 and fkY84-4 exhibited the greatest number of genes among CAZy categories, while primary active transporters contained the highest proportions of gene numbers (Table S3).

Strains fkY27-2, fkY84-1 and fkY84-4 may contain five crucial SMRs in chromosomes, and the thiopeptide region possessed two high-conserved cBGCs, but no SMRs were found in their plasmids (Figure S4A and Table S4). VFDB analysis revealed that several high-conversed VGs, including *acrB*, *rpoS*, *entB* and *ompA*, were detected in the genomes of strains fkY27-2, fkY84-1 and fkY84-4 (Table S5). GO and KEGG analyses of strains fkY27-2, fkY84-1 and fkY84-4 are shown in Figure S4B–G. GO analysis revealed that the most abundant subclasses were “cell” (1246, 36.2%) in cellular component (CC), “catalytic activity” (1921, 55.8%) in molecular function (MF) and “metabolic process” (1725, 50.1%) in biological process (BP) among 39 GO subclasses of strain fkY27-2, which is similar to that of strain fkY84-1 and fkY84-4 (Figure S4B–D). Crucial genes in all probiotic isolates were mainly enriched in four KEGG subclasses, including “ABC transporters”, “two-component system”, “biosynthesis of amino acids” and “carbon metabolism”, while “virion”, “virion part”, “bacterial chemotaxis” and “flagella assembly” contained lower percentages of annotated genes (Figure S4E–G). In addition, strains fkY27-2, fkY84-1 and fkY84-4 displayed no transparent hemolytic rings (Figure S4H–J). Furthermore, pivotal genes related to probiotic features in the above probiotic strains are presented in Table S6. Genes encoding stress proteins, such as lysine decarboxylase, thioredoxin, metal ABC transporter permease, cold-shock protein and aquaporin Z, may play crucial roles in environmental adaptability and resistance against adverse stressors. In addition, the annotated genes, including *dltB*, *dltD*, *eno*, *yfiQ*, *bdlA* and *srtA* are responsible for adhesion, biofilm regulation and immune modulation. These results suggested that three bacterial isolates belonged to non-hemolytic *Enterobacteria* strains with strong probiotic potentials.

### 3.5. Stress Tolerance, Nutrient Degradation Capacity and Antioxidant Activity

High stress resistance may enable probiotic isolates to survive and proliferate in the intestine within the host, which is widely considered as one of the ideal features in probiotic strains. In general, pH status may vary from pH 3.5 to neutral pH in intestines of different fish species, which is significantly influenced by their living surroundings [34,35]. As shown in Figure S5A–C, survival rates of strains fkY27-2, fkY84-1 and fkY84-4 were above 70% in culture with pH 4.5 during monitoring periods. In contrast, survival rates of strains fkY27-2, fkY84-1 and fkY84-4 increased from approximately 20% to 70% in culture with pH 3.5 from 3 h to 24 h. When pH dropped to 2.5, the bacterial growth rates were significantly inhibited and their survival rates maintained below 20%.

Bile salt contributes to nutrient absorption, maintenance of acid–base balance and growth inhibition of invasive pathogens [36,37] whose 0.3% concentration can play a critical role in the bacterial screening of tolerant probiotics [38]. In Figure S5D–F, all probiotic isolates displayed high survival rates (>80%) in culture with various doses of bile acid from 3 h to 12 h. In addition, survival rates of strains fkY27-2, fkY84-1 and fkY84-4 decreased from approximately 89% to 69% after 24 h, when doses of bile salt increased from 0.1% to 0.5%.

LZM belongs to the group of functional peptides that can directly participate in the immune defense against invasive pathogens, which is broadly expressed in body surface, skin and intestine of teleost fish [39]. Although the variation in LZM activity may rely on the difference in cell wall integrity and layers [40], probiotic bacteria can generally display a high resistance to LZM exposure [41]. In Figure S5G–I, strains fkY27-2, fkY84-1 and fkY84-4 displayed high survival rates (>80%) in culture with various LZM doses from 3 h to 24 h. This result suggested that strains fkY27-2, fkY84-1 and fkY84-4 belonged to LZM-tolerant strains.

Osmotic pressure may alleviate cell viability and desirable traits of probiotic isolates, while strains that can grow in a salinity range of 0.5 to 20.0 g/L may enhance their application ranges [42,43]. In Figure S5J–L, survival rates of strains fkY27-2, fkY84-1 and fkY84-4 were above 80% in culture with 2.0% NaCl salinity during monitoring periods. When NaCl salinity increased to 4.0% and 6.0%, all probiotic strains exhibited the gradual decrease in survival rates with the values ranging from approximately 35% to 80%.

In Figure S5M, the changed levels of resistance abilities were calculated by PCA. Most of the resistance abilities in strains fkY27-2, fkY84-1 and fkY84-4 were mainly located in the secondary quadrants, while the resistance ability against pH 2.5 was negatively correlated with that of 4.0% NaCl, 6.0% NaCl, 100 μg/mL LZM and pH 4.5. Furthermore, in vitro decomposing ability of starch, lipid and protein by probiotic supernatants are investigated in Table S7. The supernatant of strain fkY27-2 exhibited the highest AMS activity among probiotic isolates, accompanied by strain fk84-4, while the highest LPS and PRS activity was observed in supernatant of strain fkY84-1.

In order to investigate the antioxidant capacity, strains fkY27-2, fkY84-1 and fkY84-4 were subjected to the exposure to TBHP and H_2_O_2_. DPPHS ability maintained above 40% in strains fkY27-2, fkY84-1 and fkY84-4 following TBHP exposure, and the highest level was observed in strain fkY27-2 at 32 h post exposure (Figure S6A). In Figure S6B, DPPHS ability ranged from approximately 30% to 50% in probiotic potentials at 8 h after H_2_O_2_ exposure and then increased to the highest level (>60%) at 16 h. Strain fkY84-1 maintained the highest DPPHS ability at 32 h following H_2_O_2_ exposure, while DPPHS ability of strains fkY27-2 and fkY84-4 decreased to approximately 35%. SARS ability ranged from approximately 60% to 70% in strains fkY27-2, fkY84-1 and fkY84-4 during 32 h TBHP exposure (Figure S6C). In contrast, strain fkY84-1 exhibited the highest SARS ability with the value of above 75% following H_2_O_2_ exposure, while a dramatic decline of SARS ability was detected in strains fkY27-2 and fkY84-4 after 32 h exposure to H_2_O_2_ (Figure S6D). In Figure S6E, the changed levels of probiotic properties were calculated by PCA. The probiotic values, including SARS ability, DPPHS ability, BFA and BI, were placed in the secondary quadrants, which displayed horizontal mirror symmetry with CAg. In contrast, hydrophobicity values were negatively correlated with that of CAg. Based on the characteristics of PCA ellipses, strain fkY84-1 may display higher levels of antioxidant activities and BI performances, while strain fkY84-4 may exhibit a stronger CAg ability. These results reveal the similarity and difference in probiotic characteristics in above probiotic potentials, providing valuable insights for further probiotic application.

### 3.6. Pathogenic Antagonism of Probiotic Isolates

**Figure 5:**
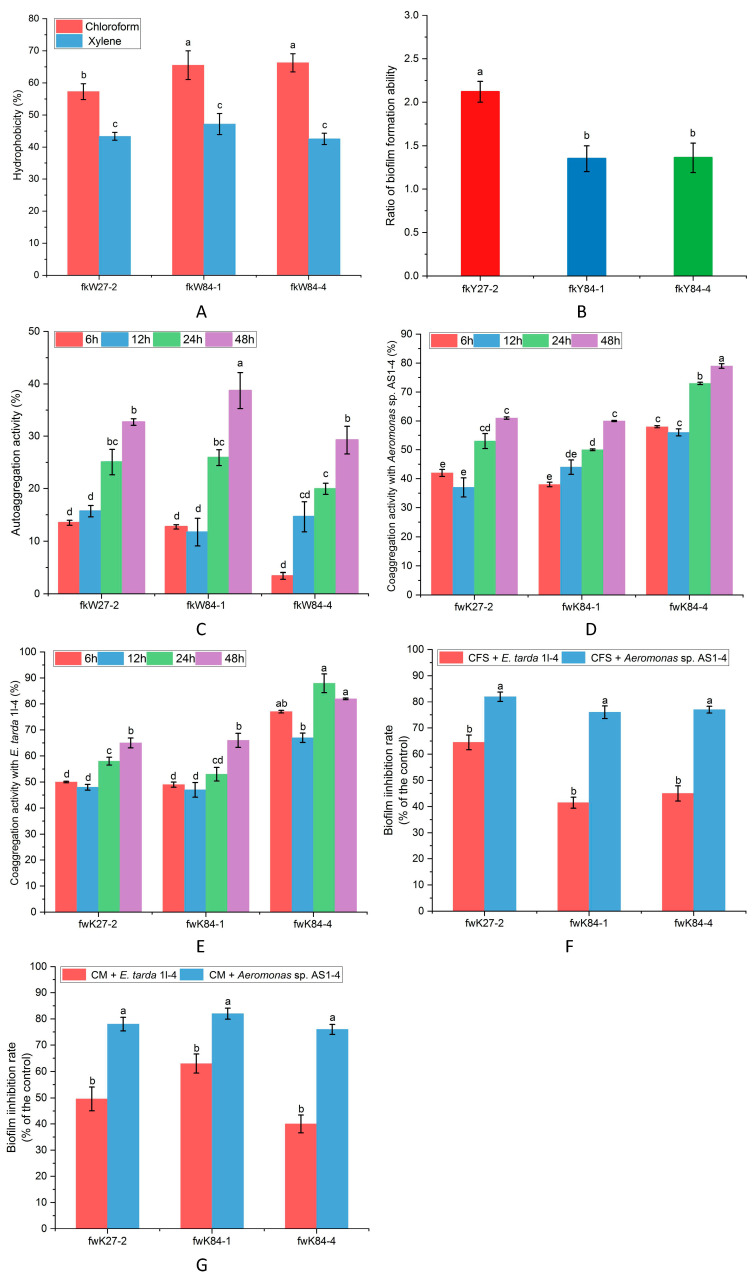
Assessment of antibacterial potential. (**A**) Cell surface hydrophobicity of probiotic isolates. (**B**) BFA ratio of probiotic isolates. (**C**) AAg percentage of probiotic isolates at different times (6 h, 12 h, 24 h and 48 h). (**D**,**E**) CAg percentage of probiotic isolates with fish pathogens at different times (6 h, 12 h, 24 h and 48 h). (**F**,**G**) BI performance of probiotic isolates. The calculated data (mean ± SD) with different letters were significantly different (*p* < 0.05) among the groups. This experiment was conducted with three biological replicates.

As depicted in Figure 5A, strains fkY27-2, fkY84-1 and fkY84-4 exhibited stronger hydrophobicity towards chloroform with a maximum value of 57.3%, 65.5% and 66.3%, while lower hydrophobicity towards xylene was observed in above strain isolates. In Figure 5B, strain fkY27-2 showed a higher OD_570_ value compared with that of strains fkY84-1 and fkY84-4, suggesting that strain fkY27-2 exhibited a higher BFA potential among bacterial isolates. Strain fkW84-1 exhibited the highest AAg ability at 48 h in comparison with that of strains fkY27-2 and fkW84-4 (Figure 5C). In contrast, strain fkW84-4 exhibited the highest CAg activities with *Aeromonas* sp. AS1-4 and *E. tarda* 1l-4 compared with that of strains fkW27-2 and fkW84-1 (Figure 5D,E). After that, CFS and CM of above probiotic isolates were collocated to validate BI ability in vitro. CFS and CM could significantly inhibit the pathogenic BFA of *Aeromonas* sp. AS1-4 and *E. tarda* 1l-4. Moreover, both CFS and CM of strains fkW27-2, fkW84-1 and fkW84-4 showed the high inhibition rates (>80%) to BFA of *Aeromonas* sp. AS1-4, while their inhibition rates to BFA of *E. tarda* 1l-4 may range from approximately 30% to 65% (Figure 5F,G).

## 4. Discussion

Among the major freshwater pathogens, *Aeromonas* strains stand out for their capacity to inflict severe diseases on aquatic animals, which may commonly provoke distressing pathological syndromes and elicit detrimental effects on their survival [44]. Our earlier transcriptomic investigation discovered that *Aeromonas* sp. AS1-4 infection was able to induce abnormal glycogen deposition and exhibit a profound effect on the insulin signaling pathway and steroid biosynthesis in TCFs [22]. In this study, we further investigate the metabolic features in TCFs infected with *Aeromonas* sp. AS1-4 and explore the multifunctional effect of probiotic isolates in efficiently inhibiting the infectious occurrence.

Fish are equipped with sophisticated complement cascades, which are vital for immune surveillance in response to invading bacteria [45]. The liver–spleen axis denotes the bidirectional interaction among immune modulation, infectious events and metabolic reactions in liver and spleen, which can orchestrate the elimination of apoptotic cells, govern the development and activation of immune cells as well as boost antibody synthesis [46]. Nevertheless, severe bacterial infection can provoke oxidative imbalance, promote lipid peroxidation as well as disrupt cellular redox equilibrium [47]. Fish rely on antioxidant enzymes and bioactive compounds to counteract cytokine toxicity, but excessive oxidative stress may exhaust their protective capacity [48]. Furthermore, excessive levels of stress-induced ROS can undermine host immune defense, disrupt metabolic regulation and hinder bacterial elimination [49]. In this investigation, the distinct expression profiles of immune-related genes were noted in the liver and spleen of TCFs after strain AS1-4 infection, along with a significant decrease in antioxidant capacity. Furthermore, metabolomic assay was employed to explore metabolic manifestations in the liver of TCFs post infection. The vast proportion of KEGG signals were related to amino acid metabolism, while most DMs were enriched in ABC transporters. Typically, ABC transporters possessing high-conserved ATP-binding domains can directly bind to ATP and use the energy to mediate the transport of a variety of molecular substances across cellular membranes, which are involved in the metabolic balance of fatty acids, amino acids and carbohydrates [50], whereas deficiency of ABC transporters may cause a wide range of diseases [51]. In addition, amino acids, along with fatty acids, are considered as optimal energy sources, while their deficiency may undermine immune function and increase the susceptibility to infectious diseases [52]. Among essential amino acids, branch chain amino acids (BCAAs), such as isoleucine, generate acetyl-CoA precursors for mTOR-dependent immune stimulation while promoting immune cell proliferation to strengthen host antimicrobial activity [53]. However, the production of MSA is either derived from ethylmalonic acid via methylmalonyl-CoA mutase, or is accumulated by the metabolism of 2-methylbutyryl-CoA, which may be used as sensitive indicators of defects in the isoleucine pathway [54]. Current findings indicated that MSA accumulation may reflect the impaired amino acid metabolism and suppress the immune regulation in TCFs following strain AS1-4 infection, and thus MSA should be regarded as a metabolic indicator among identified DMs. However, the issue related to strain AS1-4 infection still requires additional research to be fully addressed.

Probiotics assume a critical role in the regulation of fish immunity by increasing the activities of immune cells and promoting the production of antimicrobial substances [55]. Additionally, probiotics are able to improve nutrient digestion and disease resistance in aquatic animals [56,57]. Genome annotation of probiotic strains may discover potential function of genes associated with probiotic features, which may determine their ability to cope with adverse conditions in the intestine. In this investigation, three novel probiotic isolates belonging to non-hemolytic *Enterobacteria* strains were isolated from the intestine of TCFs. A total of 49 key genes related to probiotic features were discovered in genomes of probiotic isolates, including resistance genes. In vitro identification assay indicated that probiotic isolates could cope with various stressors. Additionally, their genomes contained only a small number of genes encoding virulence factors with very low E-values, which are likely to function in environmental adaptation instead of pathogenicity or hemolysis. Among them, OmpA exhibits a structural role in the bacterial integrity, which can provide the physical linkage between the outer membrane and peptidoglycan layer [58]. RpoS may operate as a sigma factor that can play a dual role in stress response and DNA replication [59], whereas AcrAB and Ent are responsible for ion transport [60,61]. Thus, these results suggested that strains fkY27-2, fkY84-1 and fkY84-4 may display high safety characteristics and strong stress resistance.

As commonly acknowledged, probiotic isolates possessing high levels of antioxidant activity and aggregation ability may exhibit a beneficial impact on fish health [62,63]. Hydrophobicity is one of the fundamental traits in cell surface properties of probiotic isolates, which can contribute to bacterial adherence capability [64]. High ratio of AAg activity may enable probiotic bacteria to firmly adhere to fish intestine, which is widely regarded as one of the important factors that can display health benefits by increasing probiotic adherence [65]. Furthermore, another favorable trait of probiotic isolates is their capability to aggregate with aggressive pathogens, which can rapidly suppress pathogenic colonization and inhibit their proliferation in fish [66]. Notably, BFA refers to the microbial growing states that can allow microorganisms to modulate interspecific communication by forming a sessile community [67]. Therefore, restriction of pathogenic BFA ability may strongly reduce colonization and alleviate pathogenicity within the host [68,69]. In contrast, probiotic BFA can promote their long-term permanence in host intestinal mucosa, which may attenuate pathogenic colonization and alleviate infectious processes [70]. Our findings indicated that probiotic isolates displaying high hydrophobicity can strongly coaggregate with *Aeromonas* sp. AS1-4 and *E. tarda* 1l-4 and inhibit their pathogenic BFA activities. The suppressive activities of strains fkY27-2, fkY84-1 and fkY84-4 against fish pathogens were similar to that of *Bacillus* sp. A2 [71]. Meanwhile, some severe pathological features may gradually deteriorate and result in fatal consequences, which are largely due to a large quantity of ROS production during pathogenic infection [72]. In this case, recent findings suggest that probiotic strains, including *Lactobacillus acidophilus* DSMZ 23033 and *Lactobacillus brevis* DSMZ 23034, can significantly mitigate excessive amounts of ROS generation and contribute to the control of several oxidative stress-induced diseases [73]. In this study, strains fkY27-2, fkY84-1 and fkY84-4 displayed high antioxidant capability against DPPH radical and superoxide anion radical, which was similar to those of *Lactobacillus* strains [74]. In addition, some probiotic strains can also rapidly decompose complex macronutrients, which may promote the absorption ratio in the intestine within the host [75]. In this investigation, supernatants of probiotic isolates exhibited high decomposing activity to starches, amino acids and lipids, which were similar to that of *Lactobacillus* sp. PSC101 [76]. These results suggested that TCF-derived *Enterobacteria* isolates exhibited a variety of probiotic characteristics, which could be used as probiotic application to prevent pathogenic infection during aquaculture processes.

## 5. Conclusions

In summary, notable enzymatic changes and dramatic changes in gene expressions were recorded in TCFs infected with *Aeromonas* sp. AS1-4. Additionally, strain AS1-4 infection may have a significant influence on amino acid metabolism in TCFs, with MSA identified as the pivotal biomarker. Then, three TCF-derived *Enterobacter* strains showed robust safety characteristics, nutrient decomposing ability and stress resistance, with strong hydrophobicity and BFA. Additionally, all probiotic isolates displayed in vitro coaggregation with fish pathogens and exhibited potent antioxidant activity against various free radicals. Our results unraveled the molecular and pathological characteristics in TCFs infected with *Aeromonas* sp. AS1-4 and in vitro probiotic identification of TCF-derived *Enterobacter* strains, which may highlight practical references for disease prevention and probiotic utilization in aquaculture.

## Data Availability

The datasets used and/or analyzed during the current study are available from the corresponding author on reasonable request.

## Supplementary Materials

The following supporting information can be downloaded at: https://www.mdpi.com/article/10.3390/biology14111485/s1, Figure S1: Gene expressions in TCFs upon-infection; Figure S2: Metabolic characteristics, model prediction and expressions of pivotal metabolic genes in liver of TCFs after strain AS1-4 infection; Figure S3: Characterization of bacterial isolates; Figure S4: Gene characteristics and hemolytic analyses of probiotic isolates; Figure S5: Stress resistance analysis; Figure S6: Antioxidant activity assessment of probiotic strains. Table S1: Primers used in this study for gene expression detection; Table S2: Genome characteristics of bacterial isolates; Table S3: Gene Percentages for CAZy and TCDB families; Table S4: Secondary metabolite regions (SMRs) in strain AS1-4 genome; Table S5: Identification of virulence genes; Table S6: Genes associated with important probiotic features in strain AS1-4 genome; Table S7: Evaluation of digestive enzyme activity in probiotic supernatants.
