# Supplementary material for: Triploid Cyprinid Fish (TCF) Under Aeromonas sp. AS1-4 Infection: Metabolite Characteristics and In Vitro Assessment of Probiotic Potentials of Intestinal Enterobacter Strains
Source: Biology (Basel). 2025 Oct 24;14(11):1485. doi: 10.3390/biology14111485 (PMC12650594; doi:10.3390/biology14111485)
Supplement: Supplementary file 1 [file biology-14-01485-s001.zip › biology-3894847-supplementary/Figure S1.pdf]

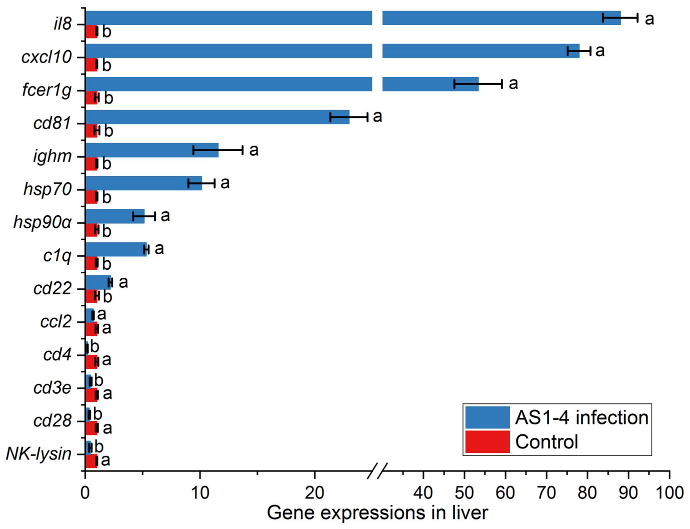

Figure S1A

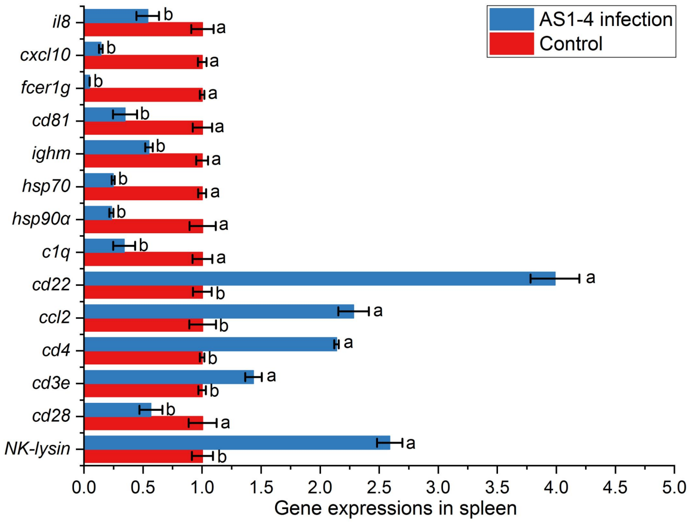

Figure S1B

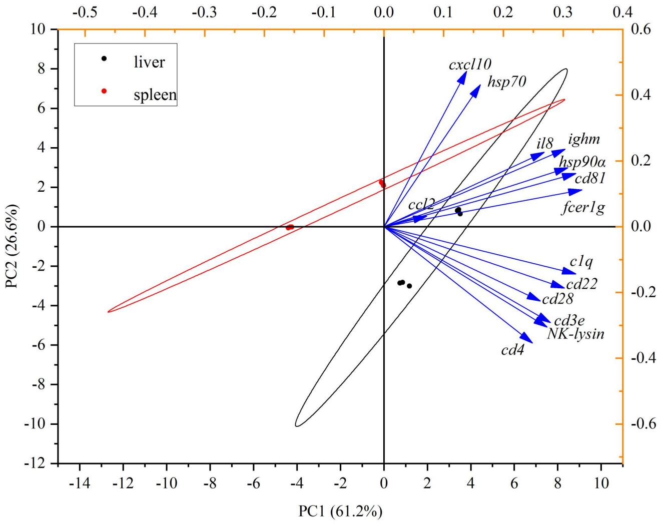

Figure S1C

Figure S1. Gene expressions in TCFs upon-infection. (A-B) Gene expressions in liver and spleen. Relative gene expression levels of target genes were normalized to 18S rRNA, and results were expressed as ratio of target gene/18S rRNA. (C) PCA analysis of gene expressions. The

calculated data (mean ± SD) with different letters were significantly different ($p < 0.05$) among the groups. The experiments contained three biological repeats.
