# Supplementary material for: Triploid Cyprinid Fish (TCF) Under Aeromonas sp. AS1-4 Infection: Metabolite Characteristics and In Vitro Assessment of Probiotic Potentials of Intestinal Enterobacter Strains
Source: Biology (Basel). 2025 Oct 24;14(11):1485. doi: 10.3390/biology14111485 (PMC12650594; doi:10.3390/biology14111485)
Supplement: Supplementary file 1 [file biology-14-01485-s001.zip › biology-3894847-supplementary/Figure S2.pdf]

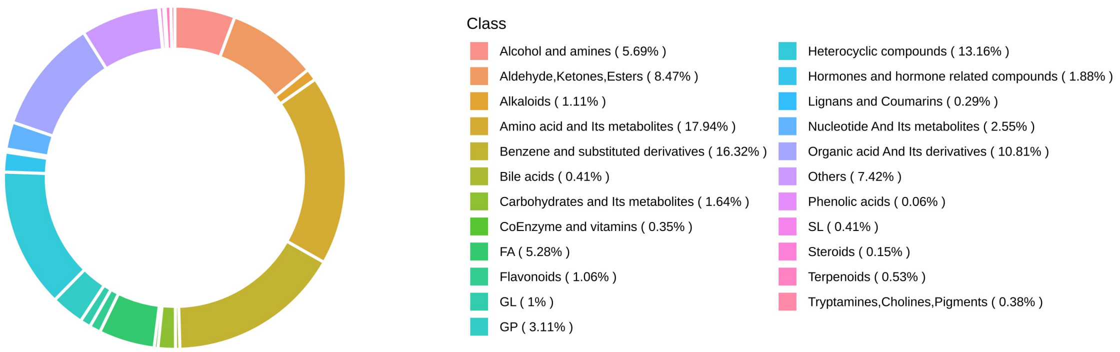

Figure S2A

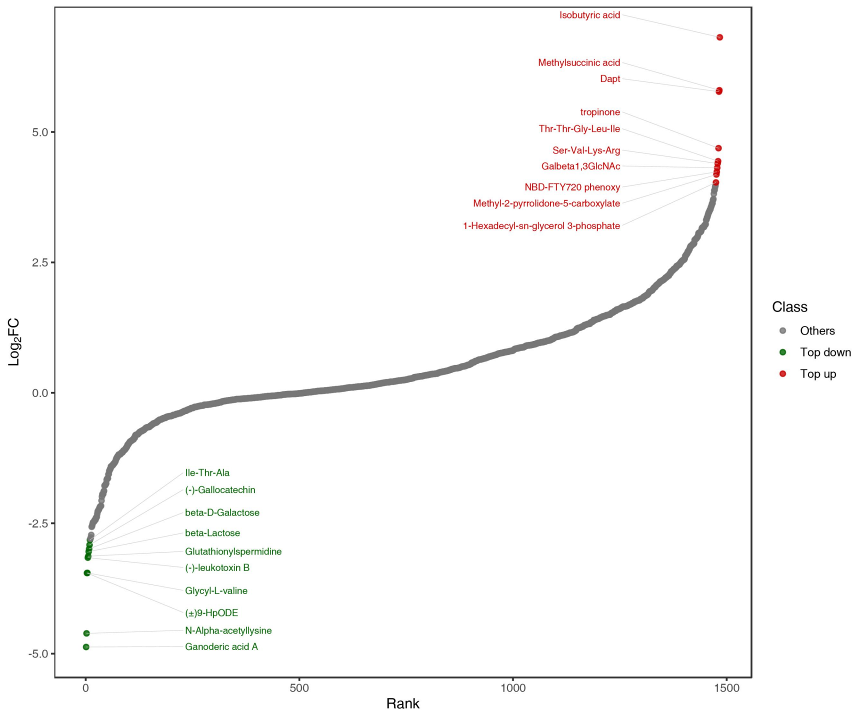

Figure S2B

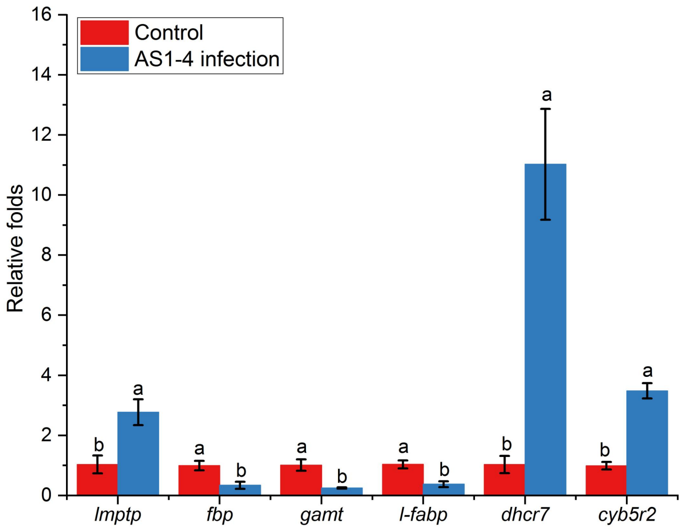

Figure S2C

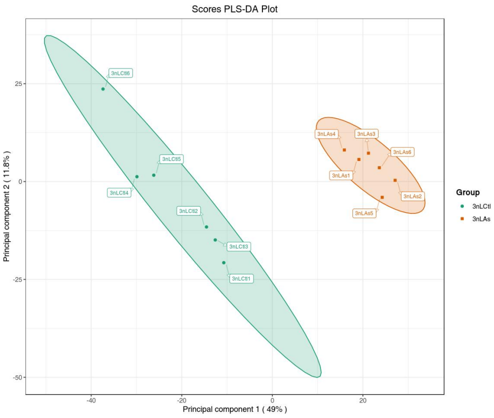

Figure S2D

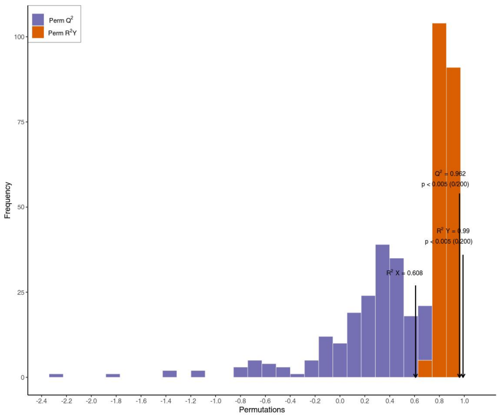

Figure S2E

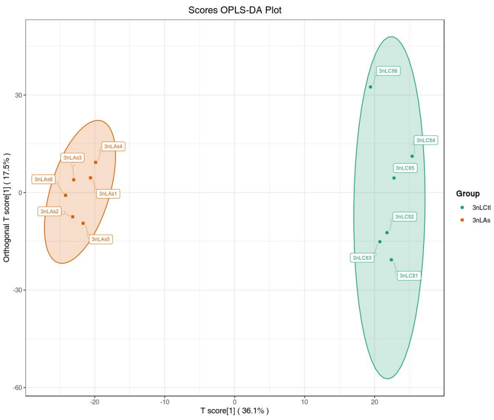

Figure S2F

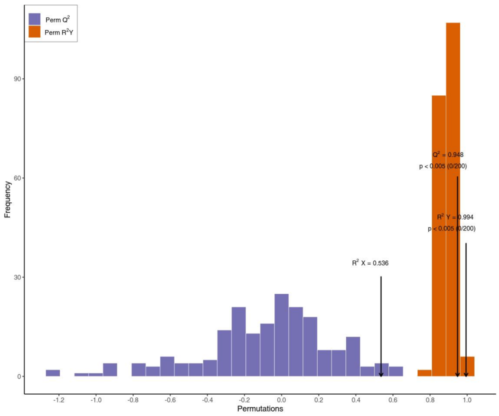

Figure S2G

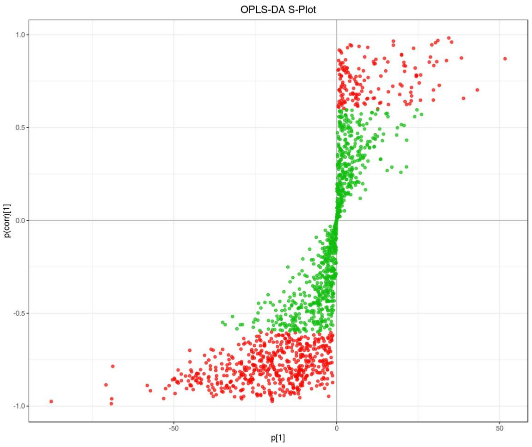

Figure S2H

Figure S2. Metabolic characteristics, model prediction and expressions of pivotal metabolic genes in liver of TCFs after strain AS1-4 infection. (A) Metabolic category in liver. Identified metabolites were classified into subclasses labeled from red to pink. (B) Dynamic distribution of metabolites in liver. Increased metabolites with high Log_2FC values were presented as red labels, while decreased metabolites with low Log_2FC values were presented as green labels.(C) Expression levels of metabolic genes evaluated by qPCR assay. Calculated data (mean ± SD) with different letters were significantly different ($p < 0.05$) among groups. The experiments contained three biological repeats. (D-E) PLS-DA plot and permutation plot analysis showing the separation between control groups and infection groups. (F-G) OPLS-DA plot and permutation plot analysis showing the separation between control groups and infection groups. (H) S plot of DMs by computing VIP threshold. Red spots represented DMs with VIP scores > 1, while green spots represented DMs with VIP scores < 1.
