# Supplementary material for: Triploid Cyprinid Fish (TCF) Under Aeromonas sp. AS1-4 Infection: Metabolite Characteristics and In Vitro Assessment of Probiotic Potentials of Intestinal Enterobacter Strains
Source: Biology (Basel). 2025 Oct 24;14(11):1485. doi: 10.3390/biology14111485 (PMC12650594; doi:10.3390/biology14111485)
Supplement: Supplementary file 1 [file biology-14-01485-s001.zip › biology-3894847-supplementary/Figure S3.pdf]

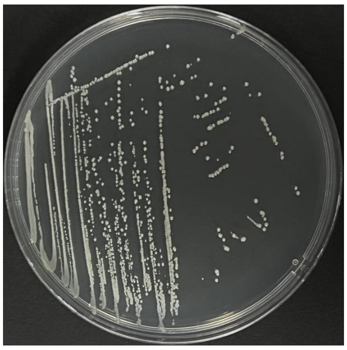

Figure S3A

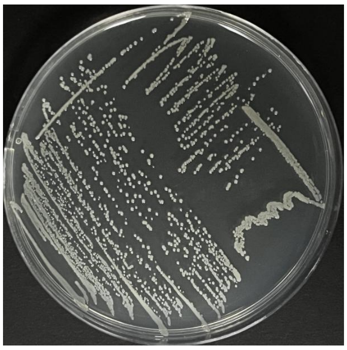

Figure S3B

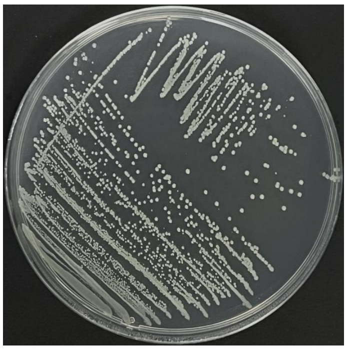

Figure S3C

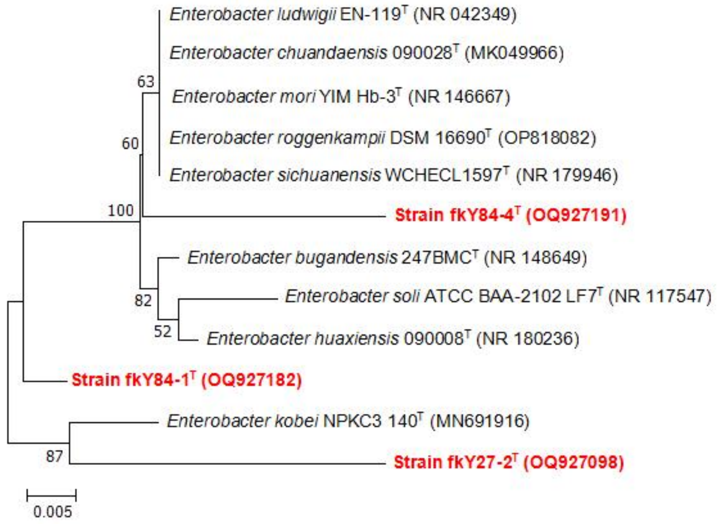

Figure S3D

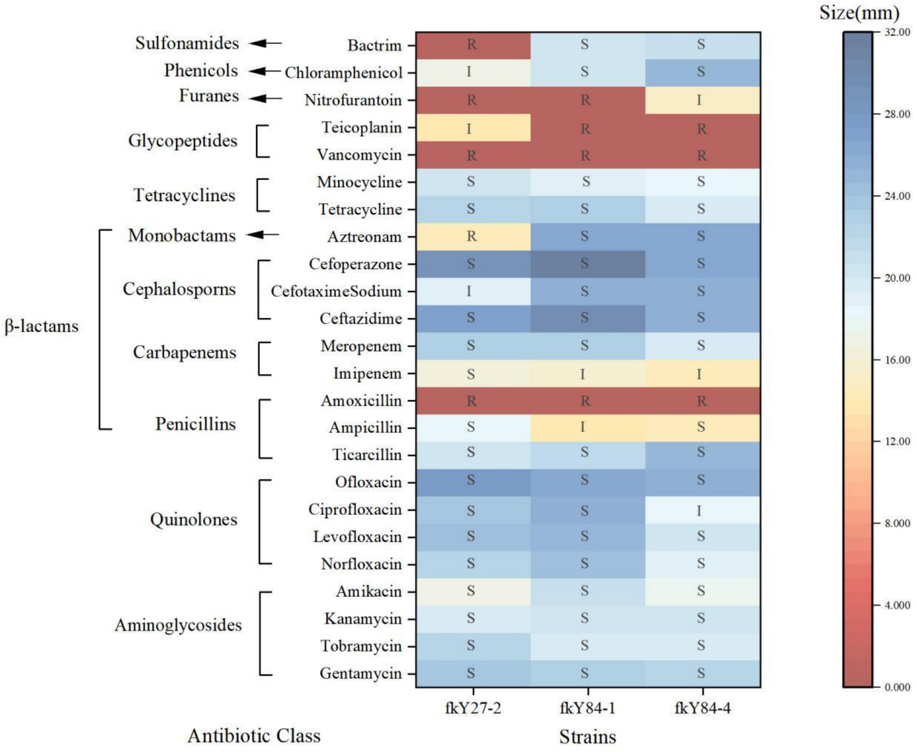

Figure S3E

Figure S3. Characterization of bacterial isolates. (A-C) Morphological determination of strains fkY27-2, fkY84-1 and Strain fkY84-4. (D) Phylogenetic tree analyses of bacterial isolates. (E) Antibiotic resistance of bacterial isolates. This experiment was conducted with three biological replicates.
