# Supplementary material for: Triploid Cyprinid Fish (TCF) Under Aeromonas sp. AS1-4 Infection: Metabolite Characteristics and In Vitro Assessment of Probiotic Potentials of Intestinal Enterobacter Strains
Source: Biology (Basel). 2025 Oct 24;14(11):1485. doi: 10.3390/biology14111485 (PMC12650594; doi:10.3390/biology14111485)
Supplement: Supplementary file 1 [file biology-14-01485-s001.zip › biology-3894847-supplementary/Figure S4.pdf]

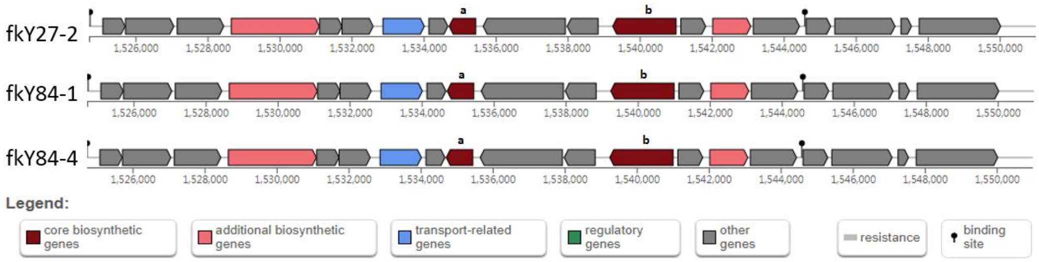

Figure S4A

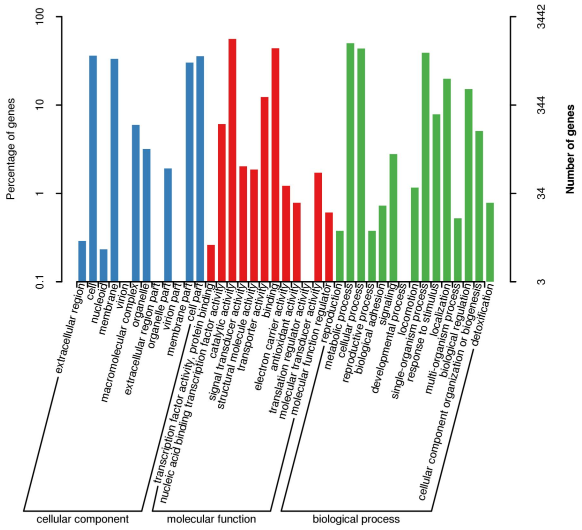

Figure S4B

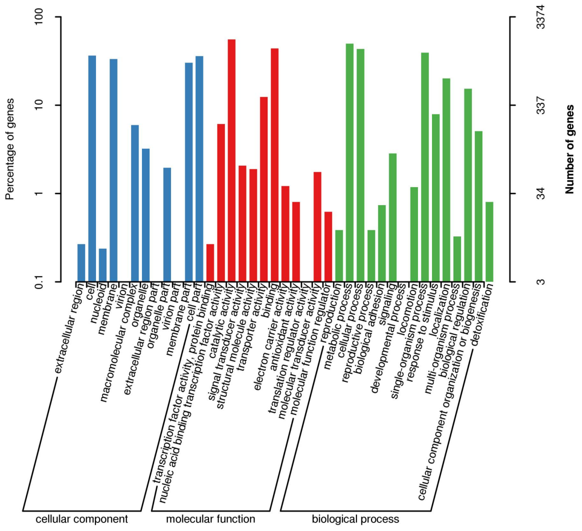

Figure S4C

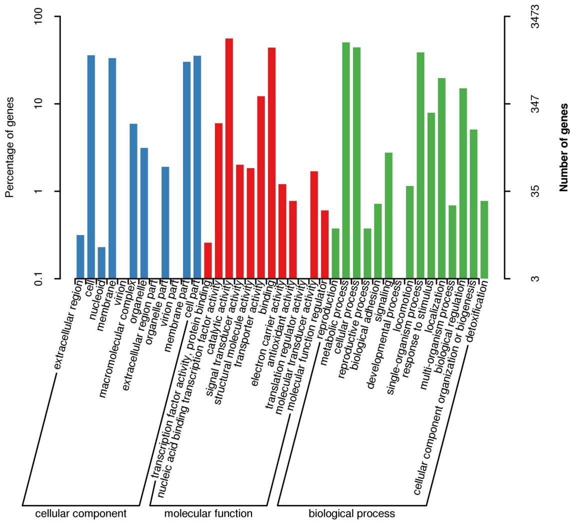

Figure S4D

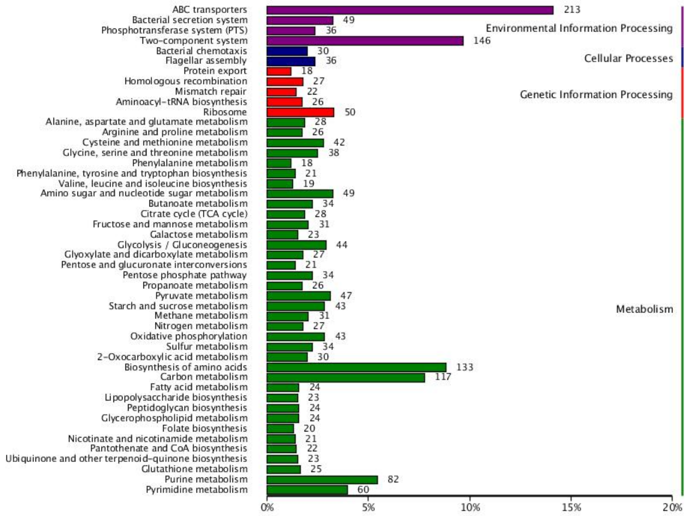

Figure S4E

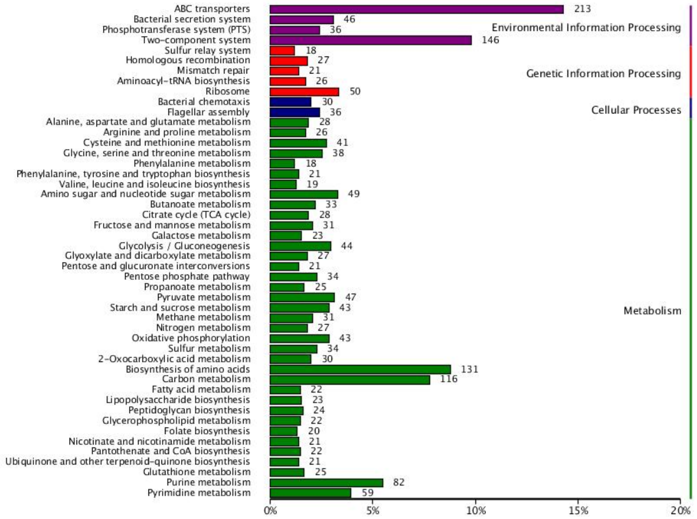

Figure S4F

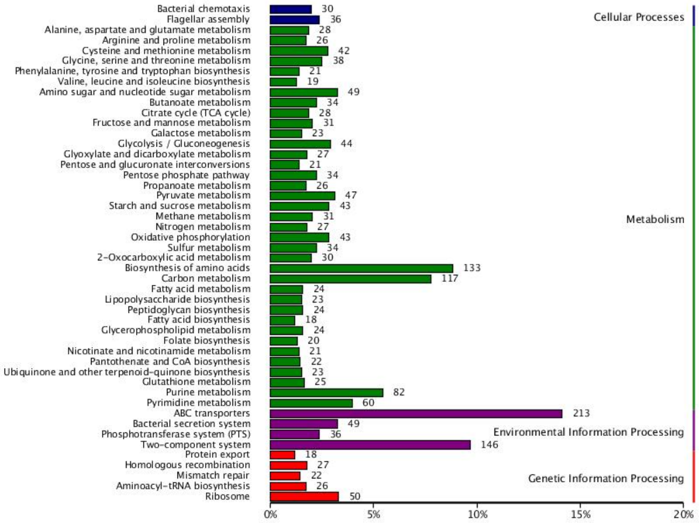

Figure S4G

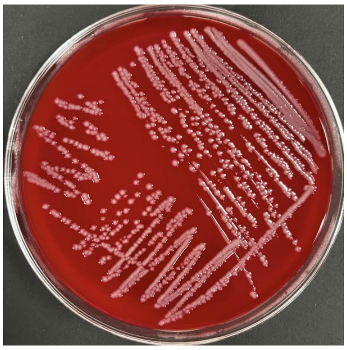

Figure S4H

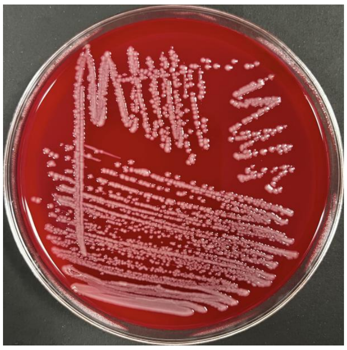

Figure S4I

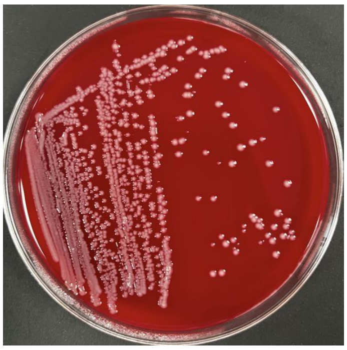

Figure S4J

Figure S4. Gene characteristics and hemolytic analyses of probiotic isolates. (A) Classification of gene clusters in thiopeptide region. (a) pyruvate formate lyase 1-activating protein and (b) 30S ribosomal protein S12 methylthiotransferase accessory factor YcaO. (B-D) GO analyses.(E-G) KEGG analyses. (H-J) Hemolytic determination of probiotic isolates.
