# Supplementary material for: Triploid Cyprinid Fish (TCF) Under Aeromonas sp. AS1-4 Infection: Metabolite Characteristics and In Vitro Assessment of Probiotic Potentials of Intestinal Enterobacter Strains
Source: Biology (Basel). 2025 Oct 24;14(11):1485. doi: 10.3390/biology14111485 (PMC12650594; doi:10.3390/biology14111485)
Supplement: Supplementary file 1 [file biology-14-01485-s001.zip › biology-3894847-supplementary/Figure S5.pdf]

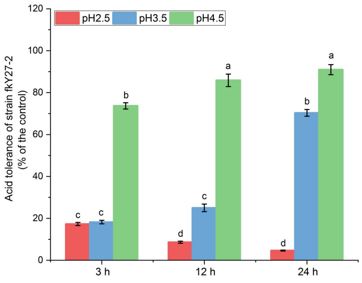

Figure S5A

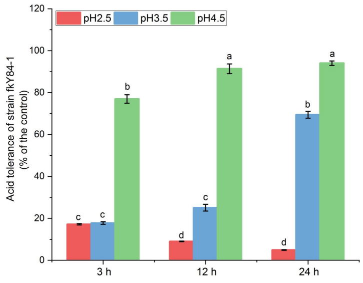

Figure S5B

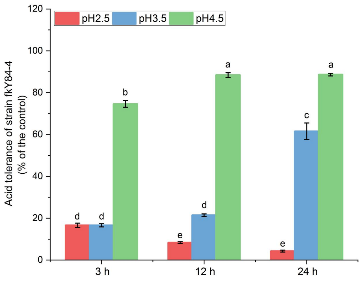

Figure S5C

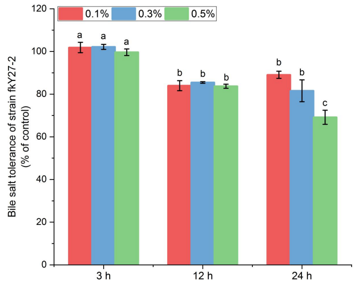

Figure S5D

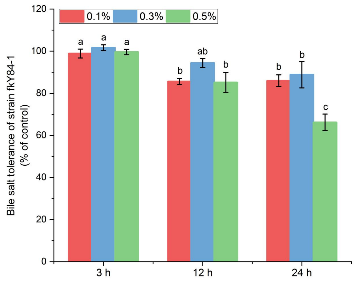

Figure S5E

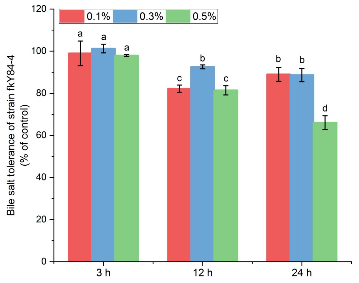

Figure S5F

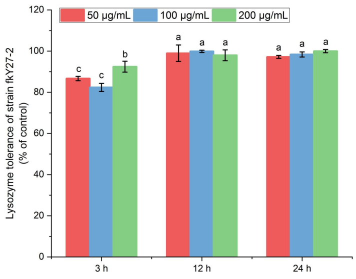

Figure S5G

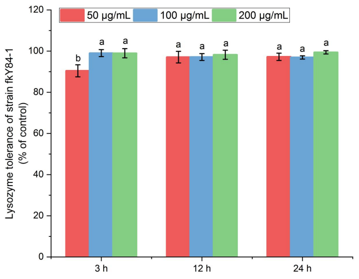

Figure S5H

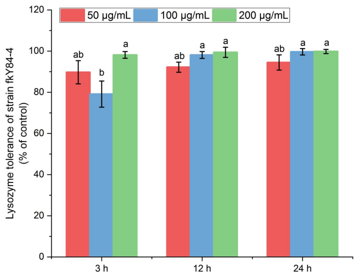

Figure S5I

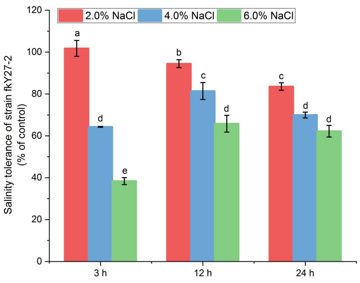

Figure S5J

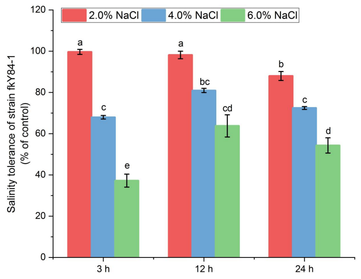

Figure S5K

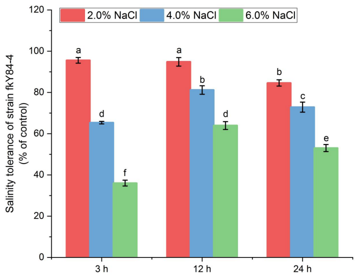

Figure S5L

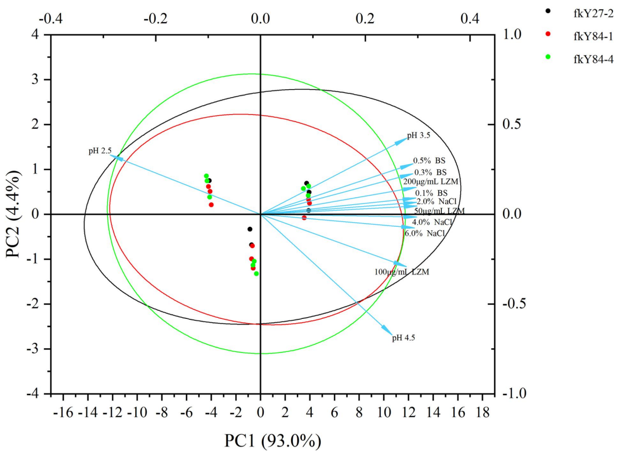

Figure S5M

Figure S5. Stress resistance analysis. (A-C) Effect of acidic condition (pH 4.5, pH 3.5 and pH 2.5) on survival rates of probiotic isolates. (D-F) Effect of bile salt (0.1%, 0.3 and 0.5%) on survival rates of probiotic isolates. (G-I) Effect of lysozyme (50, 100 and 200 μg/mL) on survival rates of probiotic isolates. (J-L) Effect of NaCl salinity (2.0%, 4.0% and 6.0%) on survival rates of probiotic isolates. (M) PCA analysis of stress resistance in probiotic isolates. LB broth without treatment was used as control group. OD_{600} values of test group, control group and blank group were showed as At, Ac and A0, respectively. Survival rate (SR) was quantified as below: SR = $(A_t - A_0)/(A_c - A_0) \times 100\%$. This experiment was conducted with three biological replicates. The calculated data (mean ± SD) with different letters were significantly different ($p < 0.05$) among the groups.
