# Supplementary material for: Triploid Cyprinid Fish (TCF) Under Aeromonas sp. AS1-4 Infection: Metabolite Characteristics and In Vitro Assessment of Probiotic Potentials of Intestinal Enterobacter Strains
Source: Biology (Basel). 2025 Oct 24;14(11):1485. doi: 10.3390/biology14111485 (PMC12650594; doi:10.3390/biology14111485)
Supplement: Supplementary file 1 [file biology-14-01485-s001.zip › biology-3894847-supplementary/Figure S6.pdf]

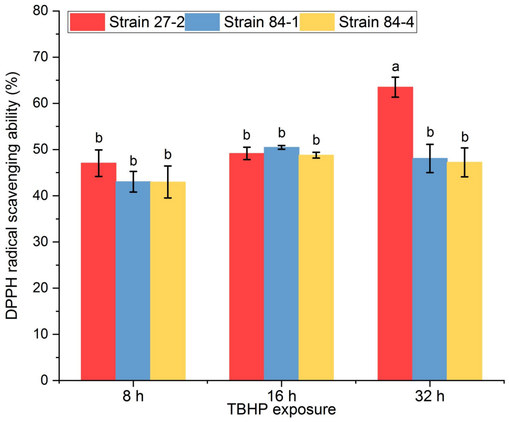

Figure S6A

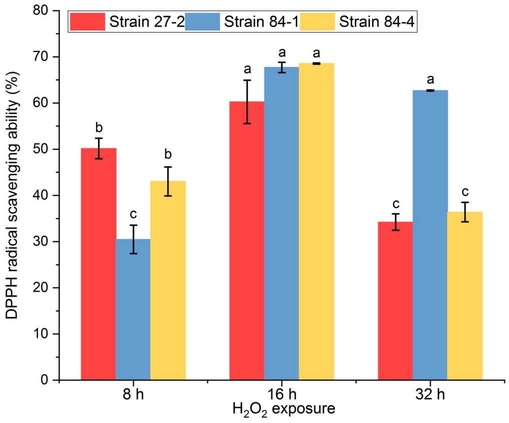

Figure S6B

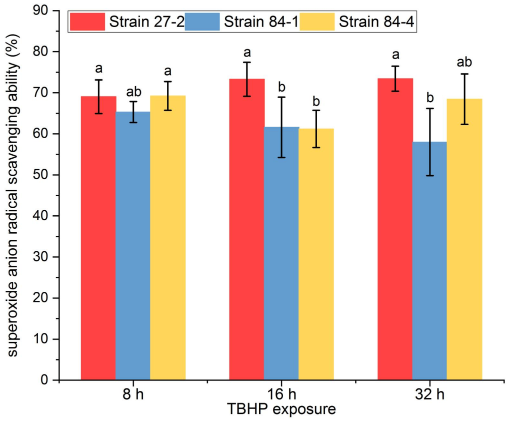

Figure S6C

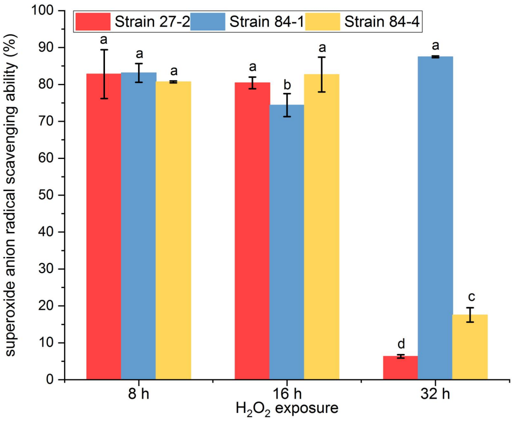

Figure S6D

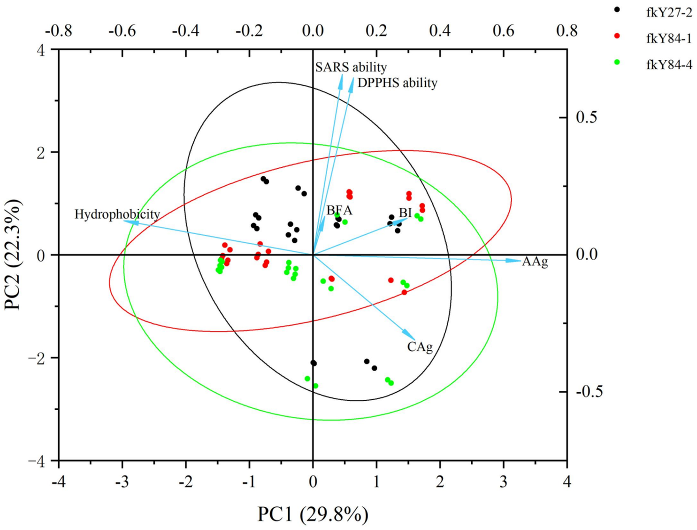

Figure S6E

Figure S6. Antioxidant activity assessment of probiotic strains. (A-B) DPPHS ability under TBHP exposure or H_2O_2 exposure. OD absorbances were measured at 517 nm. (C-D) SARS ability under TBHP exposure or H_2O_2 exposure. OD absorbances were measured at 530 nm. (E) PCA analysis of probiotic traits related to antipathogenic and antioxidant ability. FRS ability was calculated as below: FRS activity = $[(1-(A_t/A_c)]\times100\%$. The calculated data (mean ± SD) with different letters were significantly different (p < 0.05) among the groups. This experiment was conducted with three biological replicates.
