# Supplementary material for: Triploid Cyprinid Fish (TCF) Under Aeromonas sp. AS1-4 Infection: Metabolite Characteristics and In Vitro Assessment of Probiotic Potentials of Intestinal Enterobacter Strains
Source: Biology (Basel). 2025 Oct 24;14(11):1485. doi: 10.3390/biology14111485 (PMC12650594; doi:10.3390/biology14111485)
Supplement: Supplementary file 1 [file biology-14-01485-s001.zip › biology-3894847-supplementary/Table S1.pdf]

Table. S1 Primers used in this study for gene expression detection

Primer names	Sequence direction (5'→3')
RT-*18S*-F	CGGAGGTTCGAAGACGATCA
RT-*18S*-R	GAGGTTTCCCGTGTTGAGTC
RT-*lmptp*-F	CCCGTGGAAAGTCGGTATTG
RT-*lmptp*-R	TTTGGTGACCTGTCGTGCC
RT-*fbp*-F	GAGATTGTGGCAGCAGGGTA
RT-*fbp*-R	TCTGGTGGATTGAGTCAGGTTT
RT-*gamt*-F	CTTCTCAAAGGGCGAAAACTG
RT-*gamt*-R	TAGCCTCTGGAATACACCATCAT
RT-*l-fabp*-F	GAAATCCATCAAAACGGCAAC
RT-*l-fabp*-R	CCTCCTACTGTCAGGGTCTCCA
RT-*dhcr7*-F	TACCTGGGCTGGGGAGACT
RT-*dhcr7*-R	GTGGTTGGTTGAGCGGAAG
RT-*cyb5r2*-F	TGAAGATTGGAGACACCATTGAC
RT-*cyb5r2*-R	GGTTTGGCAGACAGGCGTA
RT-*NK-lysin*-F	TGGCACCAGCCCAACAA
RT-*NK-lysin*-R	GCAAGCCCAGCACAATCC
RT-*cd22*-F	ACTGGATGTATGGACAATGGC
RT-*cd22*-R	TTGCGTAGACTGCTGAACCTT
RT-*cxcl10*-F	ACTGAGTGGAGCCAGAGGTG
RT-*cxcl10*-R	AAGTGGGACTGTTGTTGATGTTC
RT-*cd28*-F	CAGAAAGATTGGAAACGGCACT
RT-*cd28*-R	AGGAAGCGACGATTAGGATGG
RT-*ccl2*-F	GCTCTGATGCTGTGGCTTCTG
RT-*ccl2*-R	CGTTTTGTTTGATACCGACTGC
RT-*cd3e*-F	ATGAGTTGAGCAGGCTGAGGG
RT-*cd3e*-R	AGGGTTCGCTGGTTTGGGA
RT-*c1q*-F	GTGGTGGCATTGATGGCAG
RT-*c1q*-R	TCGGTTTCGCAGCACAGAG
RT-*cd4*-F	GATTGCTGTAGGAGCCAGTTCT
RT-*cd4*-R	ACCCTCTTCTTCATCCGTTTG
RT-*il8*-F	CTTCCCTCCAAGCCCACA
RT-*il8*-R	TCTCAATGACCTTCTTTACCCA
RT-*fcer1g*-F	CTGATTCTCCTGCTCAACGCT
RT-*fcer1g*-R	AAATGTCCCCTTCTCGCTTCT
RT-*ighm*-F	CTTTTCGTCTGGTCTGTAATGCC
RT-*ighm*-R	CTGTGAACCTTCCCTGAACTGA
RT-*hsp70*-F	ACGAGGCAGTGGCTTATGG
RT-*hsp70*-R	GGGTCTGTTTGGTGGGGAT
RT-*cd81*-F	CAGCAACCGAGGAGCAAAA
RT-*cd81*-R	AAGGCAGCGAAACCAATCA
RT-*hsp90α*-F	AGCAGCCGATGATGGA
RT-*hsp90α*-R	GGATTTGGCGATGGTTC
