# Supplementary material for: Triploid Cyprinid Fish (TCF) Under Aeromonas sp. AS1-4 Infection: Metabolite Characteristics and In Vitro Assessment of Probiotic Potentials of Intestinal Enterobacter Strains
Source: Biology (Basel). 2025 Oct 24;14(11):1485. doi: 10.3390/biology14111485 (PMC12650594; doi:10.3390/biology14111485)
Supplement: Supplementary file 1 [file biology-14-01485-s001.zip › biology-3894847-supplementary/Table S2.pdf]

Table. S2 Genome characteristics of bacterial isolates

Strains	fkY27-2		fkY84-1		fkY84-4	
Types	Chromosome	Plasmid	Chromosome	Plasmid	Chromosome	Plasmid
Accession number	CP170092	CP170093	CP169393	CP169394	CP169395	CP169396
Topology	circular	circular	circular	linear	circular	linear
Length	4715592	118412	4674408	60531	4715592	168806
Gene sets	4312	126	4276	57	4312	185
tRNA	84	none	82	none	84	none
rRNA	25	none	24	none	25	none
5S rRNA	9	none	9	none	9	none
16S rRNA	8	none	7	none	8	none
23S rRNA	8	none	8	none	8	none
CRISPR Number	2	none	2	none	2	none
Genomic islands	8	1	8	none	8	2
Prophage	none	none	none	none	none	none
Gene cluster	5	none	5	none	5	none
Promoter predict	1100	32	1077	10	1100	47
