# Supplementary material for: Triploid Cyprinid Fish (TCF) Under Aeromonas sp. AS1-4 Infection: Metabolite Characteristics and In Vitro Assessment of Probiotic Potentials of Intestinal Enterobacter Strains
Source: Biology (Basel). 2025 Oct 24;14(11):1485. doi: 10.3390/biology14111485 (PMC12650594; doi:10.3390/biology14111485)
Supplement: Supplementary file 1 [file biology-14-01485-s001.zip › biology-3894847-supplementary/Table S3.pdf]

Table. S3 Gene Percentages for CAZy and TCDB families

category	Class	fkY27-2	fkY84-1	fkY84-4
CAZy	Carbohydrate-Binding Modules (CBM)	10.73%	10.88%	10.66%
	Auxiliary Activities (AA)	4.02%	4.08%	4.00%
	Polysaccharide Lyases (PL)	1.34%	1.36%	1.33%
	Glycosyl Transferases (GT)	23.48%	23.80%	23.33%
	Glycoside Hydrolases (GH)	47.65%	47.61%	48.00%
	Carbohydrate Esterases (CE)	12.75%	12.24%	12.66%
TCDB	Channels/Pores	12.91%	12.86%	12.81%
	Electrochemical Potential-driven Transporters	23.70%	23.25%	23.53%
	Primary Active Transporters	36.48%	35.90%	36.95%
	Group Translocators	5.33%	5.36%	5.29%
	Transmembrane Electron Carriers	2.39%	2.68%	2.37%
	Accessory Factors Involved in Transport	4.03%	4.68%	4.00%
	Incompletely Characterized Transport Systems	15.16%	15.27%	15.05%
