# Supplementary material for: Triploid Cyprinid Fish (TCF) Under Aeromonas sp. AS1-4 Infection: Metabolite Characteristics and In Vitro Assessment of Probiotic Potentials of Intestinal Enterobacter Strains
Source: Biology (Basel). 2025 Oct 24;14(11):1485. doi: 10.3390/biology14111485 (PMC12650594; doi:10.3390/biology14111485)
Supplement: Supplementary file 1 [file biology-14-01485-s001.zip › biology-3894847-supplementary/Table S4.pdf]

Table. S4 Secondary metabolite regions (SMRs) in strain AS1-4 genome

Strains	Chromosome	Plasmid	Other SMRs in Chr
fkY27-2	thiopeptide	none	NRP-metallophore, hserlactone, NI-sideropgore, arylpolyene
fkY84-1	thiopeptide	none	NRP-metallophore, hserlactone, NI-sideropgore, arylpolyene
fkY84-4	thiopeptide	none	NRP-metallophore, hserlactone, NI-sideropgore, arylpolyene
