# Supplementary material for: Triploid Cyprinid Fish (TCF) Under Aeromonas sp. AS1-4 Infection: Metabolite Characteristics and In Vitro Assessment of Probiotic Potentials of Intestinal Enterobacter Strains
Source: Biology (Basel). 2025 Oct 24;14(11):1485. doi: 10.3390/biology14111485 (PMC12650594; doi:10.3390/biology14111485)
Supplement: Supplementary file 1 [file biology-14-01485-s001.zip › biology-3894847-supplementary/Table S5.pdf]

Table. S5 Identification of virulence genes

Strain name	VFid	Identity	E-Value	Virulence factors	Matched Gene name	Matched Pathogen species
fkY27-2	VFG049144	85%	0	AcrAB	*acrB*	*Klebsiella pneumoniae* subsp. *pneumoniae* NTUH-K2044
	VFG000477	89%	0	RpoS	*rpoS*	*Salmonella enterica* subsp. *enterica serovar Typhimurium* str. LT2
	VFG048518	87%	0	Ent	*fepA*	*Klebsiella pneumoniae* subsp. *pneumoniae* NTUH-K2044
	VFG048419	86%	0	Ent	*entB*	*Klebsiella pneumoniae* subsp.*pneumoniae* NTUH-K2044
	VFG001443	87%	e^{-167}	OmpA	*ompA*	*Escherichia coli* O18:K1:H7 str. RS218
fkY84-1	VFG049144	85%	0	AcrAB	*acrB*	*Klebsiella pneumoniae* subsp. *pneumoniae* NTUH-K2044
	VFG000477	89%	0	RpoS	*rpoS*	*Salmonella enterica* subsp. *enterica serovar Typhimurium* str. LT2
	VFG048518	87%	0	Ent	*fepA*	*Klebsiella pneumoniae* subsp. *pneumoniae* NTUH-K2044
	VFG001443	87%	e^{-167}	OmpA	*ompA*	*Escherichia coli* O18:K1:H7 str. RS218
	VFG049018	86%	e^{-167}	RcsAB	*rcsB*	*Klebsiella pneumoniae* subsp. *pneumoniae* NTUH-K2044
fkY84-4	VFG049144	85%	0	AcrAB	*acrB*	*Klebsiella pneumoniae* subsp. *pneumoniae* NTUH-K2044
	VFG048518	87%	0	Ent	*fepA*	*Klebsiella pneumoniae* subsp. *pneumoniae* NTUH-K2044
	VFG048419	86%	0	Ent	*entB*	*Klebsiella pneumoniae* subsp.*pneumoniae* NTUH-K2044
	VFG001443	87%	e^{-167}	OmpA	*ompA*	*Escherichia coli* O18:K1:H7 str. RS218

Virulence genes of probiotic isolates were screened by VFDB database (identity > 85% and e-value < $1e^{-15}$)
