# Supplementary material for: Triploid Cyprinid Fish (TCF) Under Aeromonas sp. AS1-4 Infection: Metabolite Characteristics and In Vitro Assessment of Probiotic Potentials of Intestinal Enterobacter Strains
Source: Biology (Basel). 2025 Oct 24;14(11):1485. doi: 10.3390/biology14111485 (PMC12650594; doi:10.3390/biology14111485)
Supplement: Supplementary file 1 [file biology-14-01485-s001.zip › biology-3894847-supplementary/Table S6.pdf]

Table. S6 Genes associated with important probiotic features in strain AS1-4 genome

Important probiotic features	Gene	Function/Associated proteins	Gene size (bp)		
			fkY27-2	fkY84-1	fkY84-4
pH stress resistance	*atpB*	ATP synthase subunit a	816	816	816
	atpE	ATP synthase subunit c	240	240	240
	atpF	ATP synthase subunit b	471	471	471
	atpH	ATP synthase subunit delta	534	534	534
	atpA	ATP synthase subunit alpha	1542	1542	1542
	atpG	ATP synthase gamma chain	864	864	864
	atpD	ATP synthase subunit beta	1383	1383	1383
	atpC	ATP synthase epsilon chain	420	420	420
	LYD	lysine decarboxylase	2133	2133	2133
Bile salt stress resistance	*mdfA*	Multidrug transporter MdfA	1233	1233	1233
	mdfC	Multidrug transporter MdfC	3078	3078	3078
	gltK	Glutamate Aspartate transport system permease protein	1104	1104	1104
Oxidative stress resistance	*trxA*	thioredoxin	330	330	330
	msrA	peptide-methionine (S)-S-oxide reductase	642	642	642
	msrB	peptide-methionine (R)-S-oxide reductase	414	414	414
	mnH	manganese transport protein	1239	1239	1239
Heavy metal stress resistance	*corA*	magnesium transporter CorA family protein	984	984	984
	copA	COPI coat complex subunit alpha	2499	2499	2499

	znuB	metal ABC transporter permease	786	786	786
	znuC	Zinc transport system permease protein	756	756	756
Heat stress resistance	*clpB*	ATP-dependent chaperone ClpB	2574	2574	2574
	grpE	molecular chaperone GrpE (heat shock protein)	594	594	594
	hslO	heat shock protein Hsp33, C-terminal	879	879	879
	hslV	HslU-HslV peptidase proteolytic subunit 1	531	531	531
	hslU	Heat shock protein HslU	1335	1335	1335
	dnaK	Chaperone DnaK	1914	1914	1914
	dnaJ	Heat shock protein DnaJ	1146	1146	1146
Cold stress resistance	*cspC*	cold-shock protein CspC	210	210	210
Osmotic stress resistance	*mscL*	Large-conductance mechanosensitive channel	411	411	411
	aqpZ	Aquaporin Z	696	696	696
	osmY	Osmotically inducible protein OsmY	432	432	432
	osmC	Osmotically inducible protein C	429	429	429
	osmB	Osmotically inducible lipoprotein B	219	219	219
	osmE	Osmotically inducible lipoprotein	342	342	342
	gufp	Glycerol uptake facilitator protein	846	846	846
Immunomodulation	*dltB*	D-alanyl-lipoteichoic acid biosynthesis protein DltB	1119	1119	1119
	dltD	D-alanyl-lipoteichoic acid biosynthesis protein DltD	1158	1158	1158
Adhesion and aggregation	*dltD*	D-alanine transfer protein	1159	1159	1159
	slpA	FKBP-type peptidyl-prolyl cis-trans isomerase	450	450	450

	mntA	Manganese-binding lipoprotein MntA	960	960	960
Biofilm regulatory genes	*luxS*	S-ribosylhomocysteine lyase	516	516	516
	rbsB	Ribose import binding protein	942	942	942
	rbsA	Ribose import ATP-binding protein	1506	1506	1506
	rbsC	Ribose import permease protein	1164	1164	1164
	dnaK	molecular chaperone	1914	1914	1914
	vanY	D-alanyl-D-alanine carboxypeptidase	576	576	576
	pstS	Phosphate-binding protein	1041	1041	1041
	degP	Periplasmic serine endoprotease	1437	1437	1437
	yajC	Sec translocon accessory complex subunit YajC	333	333	333
