# Supplementary material for: Triploid Cyprinid Fish (TCF) Under Aeromonas sp. AS1-4 Infection: Metabolite Characteristics and In Vitro Assessment of Probiotic Potentials of Intestinal Enterobacter Strains
Source: Biology (Basel). 2025 Oct 24;14(11):1485. doi: 10.3390/biology14111485 (PMC12650594; doi:10.3390/biology14111485)
Supplement: Supplementary file 1 [file biology-14-01485-s001.zip › biology-3894847-supplementary/Table S7.pdf]

Table. S7 Evaluation of digestive enzyme activity in probiotic supernatants

Strains	Lipase (LPS) activity (U/mL)	Amylase (AMS) activity (U/mL)	Protease (PRS) activity (U/mL)
fkY27-2	300.99 ± 26.73^{b}	655.84 ± 100.91^{a}	7.29 ± 2.42^{c}
fkY84-1	403.96 ± 52.97^{a}	451.66 ± 17.50^{b}	42.41 ± 3.56^{a}
fkY84-4	155.45 ± 7.92^{c}	649.65 ± 118.37^{a}	32.42 ± 2.81^{b}
